# The Drosophila *Enhancer of split* Gene Complex: Architecture and Coordinate Regulation by Notch, Cohesin, and Polycomb Group Proteins

**DOI:** 10.1534/g3.113.007534

**Published:** 2013-10-01

**Authors:** Cheri A. Schaaf, Ziva Misulovin, Maria Gause, Amanda Koenig, Dale Dorsett

**Affiliations:** Edward A. Doisy Department of Biochemistry and Molecular Biology, Saint Louis University School of Medicine, Saint Louis, Missouri 63104

**Keywords:** Chromator, Nipped-B, Putzig, RNA polymerase, Suppressor of Hairless

## Abstract

The cohesin protein complex functionally interacts with Polycomb group (PcG) silencing proteins to control expression of several key developmental genes, such as the Drosophila *Enhancer of split* gene complex [E(spl)-C]. The E(spl)-C contains 12 genes that inhibit neural development. In a cell line derived from the central nervous system, cohesin and the PRC1 PcG protein complex bind and repress E (spl)-C transcription, but the repression mechanisms are unknown. The genes in the E(spl)-C are directly activated by the Notch receptor. Here we show that depletion of cohesin or PRC1 increases binding of the Notch intracellular fragment to genes in the E(spl)-C, correlating with increased transcription. The increased transcription likely reflects both direct effects of cohesin and PRC1 on RNA polymerase activity at the E(spl)-C, and increased expression of Notch ligands. By chromosome conformation capture we find that the E(spl)-C is organized into a self-interactive architectural domain that is co-extensive with the region that binds cohesin and PcG complexes. The self-interactive architecture is formed independently of cohesin or PcG proteins. We posit that the E(spl)-C architecture dictates where cohesin and PcG complexes bind and act when they are recruited by as yet unidentified factors, thereby controlling the E(spl)-C as a coordinated domain.

The cohesin protein complex, named for its role in sister chromatid cohesion and chromosome segregation, plays multiple dosage-sensitive roles in gene transcription (Chien *et al.* 2011a; Dorsett and Ström 2012; Dorsett and Merkenschlager 2013; Merkenschlager and Odom 2013). Mild disruption of cohesin activity alters gene expression, causing diverse developmental deficits in Drosophila, zebrafish, mice, and humans in the absence of any overt effects on chromatid cohesion or chromosome segregation (reviewed by Liu and Krantz 2009; Mannini *et al.* 2010; Dorsett 2011; Horsfield *et al.* 2012; Remeseiro and Losada 2013). In humans, these developmental disorders are known collectively as the cohesinopathies, and include Cornelia de Lange syndrome.

Given the ability of cohesin to encircle DNA topologically and hold sister chromatids together (Nasmyth 2011), much interest has centered on cohesin’s potential architectural roles in gene expression, such as facilitating looping between transcriptional enhancers and promoters. This interest also stems from the original discovery in a Drosophila genetic screen that sister chromatid cohesion factors facilitate long-range gene activation (Rollins *et al.* 1999). More recent genomic binding and chromosome conformation capture (3C) experiments confirm the idea that cohesin regulates gene transcription by controlling chromosome architecture. For instance, cohesin and the Nipped-B (*i.e.*, NIPBL) cohesin loading factor bind to virtually all extragenic enhancers and many active promoters, and decreases in cohesin dosage reduce enhancer-promoter looping interactions (Kagey *et al.* 2010; Chien *et al.* 2011b; Seitan *et al.* 2011; Schaaf *et al.* 2013a).

Cohesin has roles in gene transcription beyond controlling chromosome architecture. Cohesin and Nipped-B selectively bind to active gene promoters that have high levels of transcriptionally engaged but paused RNA polymerase II (Pol II) just downstream of the transcription start site (Fay *et al.* 2011; Faure *et al.* 2012; Schaaf *et al.* 2013a). At genes that are repressed by cohesin, cohesin and Nipped-B inhibit transition of paused Pol II to elongation at a step distinct from that controlled by the NELF (negative elongation factor) and DSIF (DRB sensitivity inducing factor) pausing factors (Fay *et al.* 2011). At a large fraction of active genes, cohesin and Nipped-B have the opposite effect, and stimulate transition to elongation (Fay *et al.* 2011; Schaaf *et al.* 2013a). At least part of cohesin’s stimulatory effect likely stems from facilitating enhancer-promoter interactions, which aids phosphorylation of Pol II and the pausing factors by positive transcriptional elongation factor b (P-TEFb) kinase. The evidence also suggests, however, that cohesin stimulates Pol II kinase activity independently of its role in enhancer-promoter communication because cohesin depletion simultaneously decreases the levels of phosphorylated Pol II but increases the levels of P-TEFb and Cdk12 Pol II kinases in the bodies of many active genes (Schaaf *et al.* 2013a). Cohesin also indirectly alters transcription of many active genes that don’t bind cohesin, which may arise in part from its positive regulation of the *myc* gene (Rhodes *et al.* 2010; Schaaf *et al.* 2013a), which encodes a protein that directly stimulates transcription of most active genes (Lin *et al.* 2012; Nie *et al.* 2012).

Cohesin directly facilitates binding of the PRC1 Polycomb group (PcG) complex to active genes, where PRC1 inhibits premature entry of underphosphorylated Pol II into elongation (Schaaf *et al.* 2013b). Cohesin facilitates PRC1 binding to active genes despite the absence of the PRC2 complex, which makes the histone H3 lysine 27 trimethylation (H3K27me3) mark that aids PRC1 binding to PcG-silenced genes. Indeed, cohesin depletion simultaneously decreases PRC1 binding to active genes and increases PRC1 binding to silenced genes, indicating that cohesin indirectly controls silencing by sequestering much of the available PRC1 at active genes (Schaaf *et al.* 2013b). Although cohesin is absent from the transcription units of PcG-silenced genes, it binds many polycomb response elements (PREs) that initiate and mediate silencing (Misulovin *et al.* 2008). Cohesin depletion can reduce PRE−PRE looping interactions, suggesting that although cohesin indirectly inhibits silencing by sequestering PRC1, it may simultaneously architecturally support silencing (Schaaf *et al.* 2013b). Taken together, therefore, the current data indicate that cohesin directly and indirectly controls the transcription of a majority of active and PcG-silenced genes via a combination of architectural and other mechanisms.

Several of the genes that are strongly repressed by cohesin are unusual in that they show rare extended overlaps of cohesin and the H3K27me3 mark made by PRC2 (Schaaf *et al.* 2009; Fay *et al.* 2011; Schaaf *et al.* 2013b). These genes, all of which encode key developmental transcription factors, are not fully silenced, and their transcription increases substantially upon depletion of either cohesin or PRC1. These genes do not have the cohesin-H3K27me3 state in all cell types. For example, the *invected* and *engrailed* gene complex has the cohesin-H3K27me3 state in ML-DmBG3 (BG3) cells derived from Drosophila central nervous system but not in Sg4 cells or wing discs. In Sg4 cells and anterior wing disc, the *invected-engrailed* gene complex has a PcG-silenced state with H3K27me3 and PRC1 but no cohesin, and in anterior wing disc, it is transcriptionally active and binds cohesin and PRC1 without H3K27me3 (Schaaf *et al.* 2009; Schaaf *et al.* 2013b). This raises the question of whether the cohesin-H3K27me3 state is a transition between the silenced and active states and/or a specialized state needed to restrain and hold transcription at a critical submaximal level. The roles of cohesin and PRC1 in maintaining this rare state are also unknown.

To gain further insights into the cohesin-H3K27me3 restrained state, we examined the roles of cohesin and PRC1 in controlling the architecture and expression of the *Enhancer of split* gene complex [E(spl)-C] in BG3 cells. This complex contains twelve short genes, many of which encode helix-loop-helix (HLH) DNA binding proteins that repress neural fate, and which are transcriptionally activated by the Notch receptor. In BG3 cells, the entire 50-kb complex binds cohesin and has the H3K27me3 histone modification made by the PRC2 complex (Schaaf *et al.* 2009). Surprisingly, we find that the E(spl)-C has a highly self-interactive architecture that is independent of cohesin, PRC1, and the Chromator-Putzig/Z4 (Chro-Pzg/Z4) protein complex that binds near the ends of the gene complex. Cohesin and PRC1 depletion increase binding of the Notch activator to E(spl)-C genes, which likely stems at least in part from increased expression of Notch ligand genes. Based on these and prior findings we posit that the E(spl)-C architecture determines where cohesin and PRC1 bind in the E(spl)-C when they are recruited by unknown factors, and that cohesin and PRC1 control E(spl)-C transcription through combined direct and indirect mechanisms.

## Materials and Methods

### BG3 cell culture and treatments

BG3 cells were cultured in Schneider’s media containing 10% fetal calf serum and 10 μg per mL human insulin. RNAi depletion of Rad21, Nipped-B, Ph, Pc, Chro, and Pzg/Z4 were performed with the use of double-stranded RNA as previously described (Schaaf *et al.* 2009; Fay *et al.* 2011; Schaaf *et al.* 2013a,b). Protein depletion of all proteins except Chro was evaluated by western blotting via the use of previously described Rad21, Nipped-B, Ph, and Pc antibodies (Gause *et al.* 2008; Schaaf *et al.* 2009; Fay *et al.* 2011; Schaaf *et al.* 2013b). Harald Saumweber (University of Saarland) kindly provided the Pzg/Z4 antibody. To hyperactivate Notch, ethylenediamine tetraacetic acid (EDTA) was added to the culture media at a final concentration of 2 mM for 30 min. Notch processing was inhibited by addition of the DAPT (Sigma-Aldrich) γ secretase inhibitor to the culture media at a concentration of 10 μM overnight. Cells were treated with 5 μg per mL aphidicolin for 26 hr to block the cells in G1/S or with 3% dimethyl sulfoxide for 26 hr to block in G2. Fluorescence-activated cell-sorting analysis was performed to confirm the cell cycle blocks after propidium iodide staining.

### Chromatin isolation and immunoprecipitation

Chromatin was prepared and chromatin immunoprecipitation (ChIP)-chip and ChIP-quantitative polymerase chain reaction (qPCR) were performed as previously described (Misulovin *et al.* 2008; Fay *et al.* 2011). The H2Aub antibody was purchased from Cell Signaling (#8240). The H2Aub ChIP-chip data has been submitted to the GEO database (accession no. GSE49634). The Rad21 antibody was previously described (Fay *et al.* 2011). The NICD antibody (C17.9C6) was obtained from the Developmental Studies Hybridoma Bank (University of Iowa). The Su(H) antibody was purchased from Santa Cruz Biotechnology (sc-15813).

### RNA isolation and quantification

Total RNA was isolated and transcripts were quantified by reverse-transcription (RT)-qPCR as previously described (Schaaf *et al.* 2009).

### Chromosome conformation capture

3C analysis was performed as previously described (Schaaf *et al.* 2013b).

### Salivary gland polytene chromosome immunostaining

Salivary gland polytene chromosomes were fluorescently immunostained as previously described (Dorsett *et al.* 2005). The Rad21 antibodies were previously described (Gause *et al.* 2008). Kristen Johansen (Iowa State University) generously provided Chro antibody, and Harald Saumweber (University of Saarland) provided Pzg/Z4 antibody. Fluorescence intensity along stretches of salivary chromosomes was quantified using Leica software.

## Results

### Cohesin and PRC1 depletion increases Notch activator occupancy at the *Enhancer of split* gene complex in ML-DmBG3 (BG3) cells

**Figure 1:**
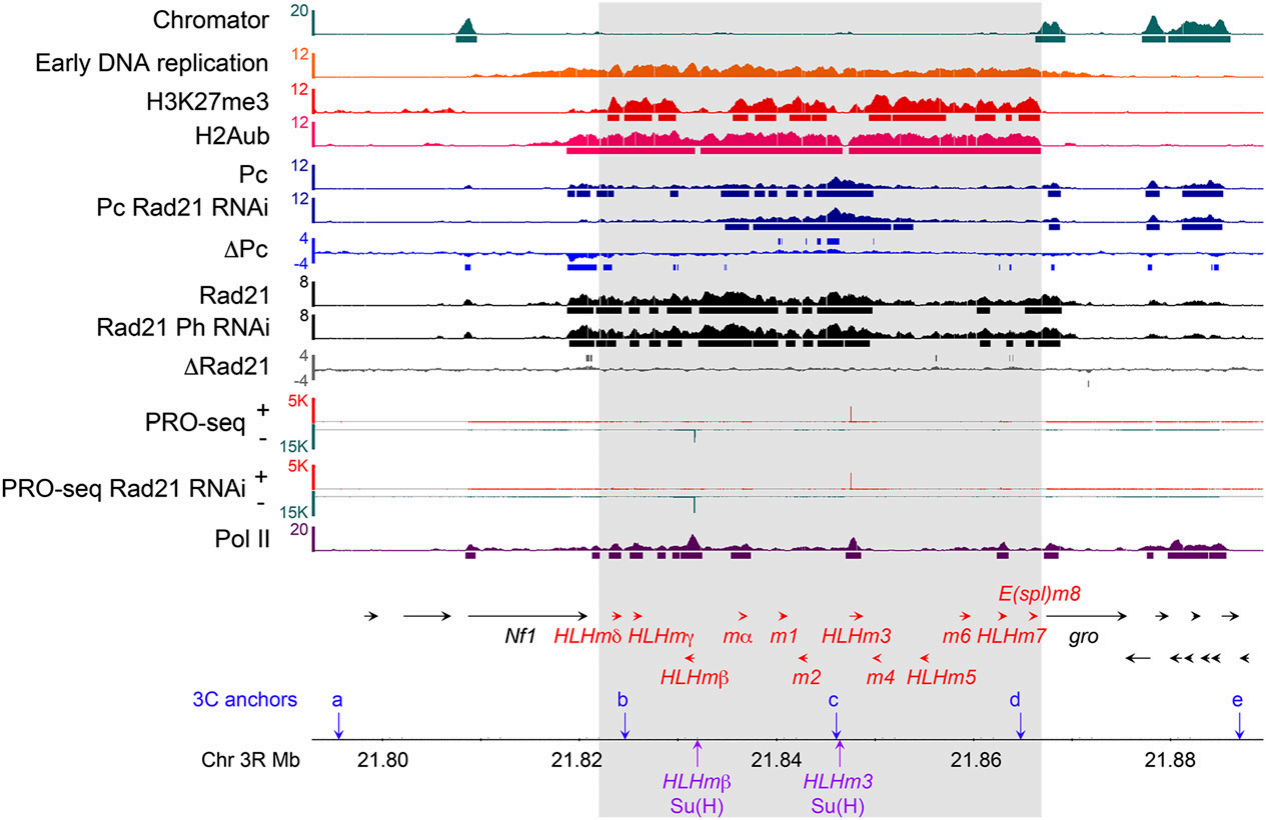
The Drosophila *Enhancer of split* gene complex [E(spl)-C]. The map of the E(spl)-C shows the 12 genes as red arrows. Flanking genes are indicated with black arrows. The locations of the five anchors (a−e) used for chromosome conformation capture (3C) are indicated with downward arrows, and the amplicons used for NICD and Su(H) ChIP-qPCR [HLHmβ Su(H), HLHm3 Su(H)] are show with upward arrows. The tracks above gene map show the ChIP-chip analysis in BG3 cells for Chromator (Chro) and early DNA replication (modENCODE Consortium *et al.* 2010), the histone H3 lysine 27 trimethylation (H3K27me3) mark made by the PRC2 PcG complex (Schwartz *et al.* 2010), the histone H2A lysine 119 mono-ubiquitination (H2Aub) modification made by the PRC1 PcG complex (this study), the Pc PRC1 subunit in control and cohesin (Rad21) depleted cells (Schaaf *et al.* 2013b), the Rad21 cohesin subunit in control and PRC1 (Ph) depleted cells (Schaaf *et al.* 2013b), and Pol II (Misulovin *et al.* 2008). The bars beneath the ChIP-chip tracks indicate binding is significant at *P* ≤ 10^−3^ using the MAT (Johnson *et al.* 2006) algorithm. The ΔPc and ΔRad21 tracks show the difference in ChIP MAT score between the Rad21 and Ph RNAi−treated and Mock control cells at each microarray feature (Schaaf *et al.* 2013b). The bars above and below the ΔPc and ΔRad21 tracks indicate where this difference is two standard deviations more or less from the mean genome-wide difference for at least three microarray features in a row (~105 bp) (Schaaf *et al.* 2013b). The PRO-seq (global run-on sequencing) tracks show the levels of transcriptionally engaged Pol II for the plus (+, red) and minus (−, green) strands (Schaaf *et al.* 2013b).

Cohesin binding overlaps the H3K27me3 silencing mark made by PRC2 complex throughout the E(spl)-C in BG3 cells (Figure 1; Misulovin *et al.* 2008; Schaaf *et al.* 2009). As predicted by the H3K27me3 pattern, the PRC1 PcG complex is also present at many locations spread throughout the E(spl)-C complex in BG3 cells (Figure 1; Schaaf *et al.* 2013b). We confirmed that PRC1 at the E(spl)-C is active by conducting genomic ChIP with tiling microarrays (ChIP-chip) for the mono-ubiquitinated histone H2A (H2Aub) modification produced by the Sce subunit of PRC1. Similar to the cohesin and H3K27me3 patterns, the H2Aub modification extends throughout the entire complex, but not into flanking regions (Figure 1).

RNAi-mediated depletion of cohesin, kollerin, or PRC1 subunits increases RNA produced by the active E(spl)-C genes by 5- to 100-fold in BG3 cells, depending on the gene and the extent of depletion (Schaaf *et al.* 2009; Fay *et al.* 2011; Schaaf *et al.* 2013a, 2013b). RNA turnover experiments show that the greater mRNA levels upon cohesin depletion are caused by increased transcription not increased RNA stability (Schaaf *et al.* 2009). The similar response of all the active E(spl)-C genes to cohesin and PRC1 depletion, and the extended cohesin binding and PcG histone marks suggests that cohesin and PRC1 regulate the E(spl)-C as a coordinated chromatin domain.

Pol II is transcriptionally engaged and paused at many of the promoters of the active E(spl)-C genes before cohesin or PRC1 depletion, indicating that the genes are poised for increased transcription (Figure 1; Schaaf *et al.* 2013a). Individual E(spl)-C genes are directly activated by the Notch receptor (Bailey and Posakony 1995). The increased E(spl)-C transcription may thus in part reflect the 2- to 5-fold increases in *Serrate* Notch ligand gene expression that occur upon cohesin or PRC1 depletion (Schaaf *et al.* 2009; Schaaf *et al.* 2013b). Cohesin and PRC1 both bind to the *Serrate* promoter region (Misulovin *et al.* 2008; Schaaf *et al.* 2013b).

**Figure 2:**
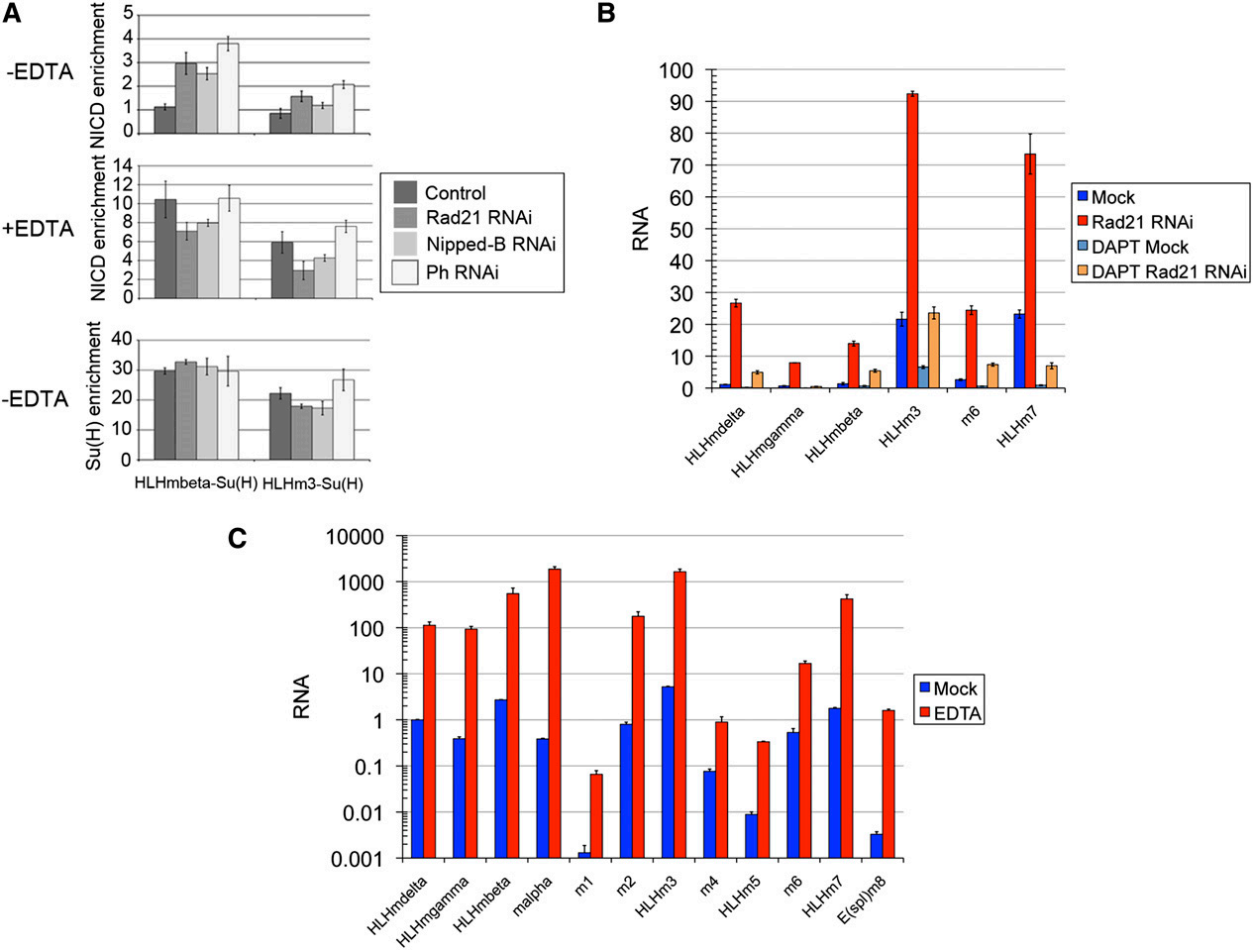
Cohesin and PRC1 restrict Notch activator binding to the E(spl)-C when Notch activation is low in BG3 cells. (A) The top panel shows the ChIP-qPCR analysis of NICD binding to the *HLHmβ* and *HLHm3* Su(H) binding sites in BG3 cells and BG3 cells depleted for Rad21 (cohesin), Nipped-B (kollerin), and Ph (PRC1) for 5 d. ChIP was performed with two to four independent chromatin preparations for each group. Enrichment of the binding site sequences is calculated relative to an empty control site on chromosome 3R. Standard errors were calculated using all biological and qPCR replicates. The middle panel shows the same NICD ChIP-qPCR analysis as in the top panel, except that Notch was activated by treating the cells with EDTA for 30 min before isolating chromatin. The cells were from the same cultures used to prepare chromatin without EDTA treatment. The bottom panel shows the ChIP-qPCR for Su(H) using the same chromatin samples used in the top panel. (B) Blocking Notch processing with the DAPT γ secretase inhibitor overnight reduces the expression of the E(spl)-C genes in control cells and cells depleted for cohesin (Rad21) for 5 d. DAPT or vehicle control was added to the cultures on the evening of the fourth day of RNAi treatment. RNA levels were quantified by RT-qPCR, normalized to *RpL32* RNA levels, and expressed relative to the *HLHmδ* RNA levels in the control Mock-treated cells. Standard errors were calculated using all qPCR replicates. The data shown is from one experiment, but similar results were obtained in multiple independent experiments. (C) Treatment of BG3 cells with EDTA for 30 min increases E(spl)-C gene transcripts 100-fold or more. Transcript levels were quantified as described for panel B, and are shown on a log_10_ scale.

Activation of Notch releases the Notch intracellular fragment (NICD) that translocates to the nucleus and interacts with the Suppressor of Hairless [Su(H)] protein bound upstream of each E(spl)-C gene. We used ChIP-qPCR to measure the levels of Su(H) and NICD at known Su(H) binding sites upstream of the *HLHmβ* and *HLHm3* genes, which show the highest levels of paused Pol II by PRO-seq, before and after cohesin and PRC1 depletion. NICD binding increased from undetectable to 3- and 1.5-fold enrichment at the *HLHmβ* and *HLHm3* Su(H) binding sites in the E(spl)-C when BG3 cells were depleted for the Rad21 cohesin subunit, and similar increases occurred when the Ph subunit of PRC1 was depleted (Figure 2A). Significant NICD binding was detected at *HLHmβ*, but not at *HLHm3* when the Nipped-B subunit of the kollerin complex that loads cohesin onto chromosomes was depleted. Su(H) binding was detected at these sites in untreated cells, and the levels were not significantly altered by cohesin, kollerin, or PRC1 depletion (Figure 2A).

We examined NICD occupancy of Su(H) binding sites upstream of other E(spl)-C genes, but it was not detectable even after cohesin or Ph depletion. The difficulty in detecting NICD at the E(spl)-C genes arises from several factors. NICD is usually present at low levels, does not directly bind DNA, and NICD bound to genes is subject to ubiquitination and degradation (Fryer *et al.* 2002, 2004). The genes where we can detect NICD, *HLHmβ* and *HLHm3*, in addition to higher levels of paused Pol II, show regions of lower H3K27me3 and H2Aub histone marks than other E(spl)-C genes (Figure 1, Supporting Information, Figure S1A). Close examination reveals significant H2Aub levels over the bodies of both of these genes, and H3K27me3 and Pc over *HLHm3* (Figure S1A). By ChIP-qPCR, these genes show a 7- to 10-fold enrichment of H3K27me3 at their promoters (Figure S1B). These observations, and the fact that cohesin and PRC1 depletion alters expression of *HLHmβ* and *HLHm3* in the same manner as it alters expression of the other E(spl)-C genes, argues that they are under the same type of regulation by cohesin and PRC1. The lowest levels of PcG histone marks in both cases are between the promoter-paused Pol II and the upstream Su(H)-binding sites, suggesting that greater Pol II and Su(H) occupancy may create a region of reduced nucleosome density (Figure S1A).

Although we did not detect NICD at any of the E(spl)-C genes examined in control cells, the basal levels of E(spl)-C transcription are Notch-dependent. Overnight treatment of BG3 cells with DAPT, a γ secretase inhibitor that blocks Notch receptor processing, reduces E(spl)-C transcripts (Figure 2B). The increases in E(spl)-C transcripts upon cohesin depletion are diminished by DAPT treatment, consistent with the idea that increased NICD occupancy contributes to the increased transcription (Figure 2B).

Treatment of Drosophila cell lines with EDTA releases the NICD fragment from the Notch receptor at the cell membrane, mimicking strong receptor activation, and greatly increases expression of Notch target genes (Krejcí and Bray 2007). EDTA treatment of BG3 cells increased multiple E(spl)-C transcripts a hundred-fold or more (Figure 2C), and NICD occupancy upstream of *HLHmβ* and *HLHm3* increased to 10-fold and 6-fold enrichment (Figure 2A). Under these conditions, depletion of Rad21, Nipped-B, or Ph did not further increase NICD occupancy (Figure 2A).

The aforementioned experiments argue that when Notch activation is low, depletion of cohesin or PRC1 increases E(spl)-C transcription by increasing the amount of NICD bound to Su(H) upstream of the individual E(spl)-C genes. This could reflect increased *Serrate* expression, combined with direct effects on NICD occupancy at the E(spl)-C. For example, cohesin and PRC1 could form a structure that restricts access of NICD to the entire gene complex, or cohesin and PRC1 bound to the E(spl)-C might also facilitate NICD degradation.

We considered the possibility that depletion of cohesin or PRC1 might have similar direct effects on E(spl)-C transcription if their binding is codependent but found that this is not the case. Cohesin interacts directly with PRC1 (Strübbe *et al.* 2011) and cohesin depletion reduces binding of PRC1 to active genes that lack H3K27me3, and increases binding of PRC1 to genes with H3K27me3 (Schaaf *et al.* 2013b). At the E(spl)-C, there is a modest decrease in Pc levels at the centromere-proximal (left) end where H3K27me3 is low, and an increase in the middle of the complex where H3K27me3 is high upon Rad21 depletion (Figure 1). There is no substantial change in Rad21 binding after Ph depletion (Figure 1). Depletion of Rad21 for several days also does not affect the levels of H3K27me3 at the several positions tested (Figure S1B).

Simultaneous depletion of Rad21 and Pc did not give a synergistic increase in E(spl)-C transcripts, even with submaximal depletions of both (Figure S2). The transcript increases upon Rad21 and Pc codepletion were either similar to the increases seen by depletion of one or the other, or slightly greater. This argues that cohesin and PRC1 together target the same step in E(spl)-C transcription, so that loss of one is equivalent to functional loss of both.

### The E(spl)-C complex has a self-interactive greater order structure independent of cohesin and PRC1

Evidence from the *invected-engrailed* gene complex, which is also co-repressed by cohesin and PRC1 in BG3 cells, but is not Notch-activated, suggests that cohesin architecturally contributes to PRC1-mediated repression by facilitating long-range looping interactions between PREs (Schaaf *et al.* 2013b). PREs initiate and maintain PcG-silencing, and PcG protein-dependent PRE−PRE interactions increase their activity (Delest *et al.* 2012; Kassis and Brown 2013). No PREs in the E(spl)-C have been identified, and we thus tested the idea that cohesin and PRC1 might facilitate looping interactions that repress E(spl)-C transcription. The idea that the E(spl)-C might have a cohesin-PRC1−dependent architecture also arose from the binding of cohesin, H3K27me3, and H2Aub throughout the entire E(spl)-C in BG3 cells, but not in the flanking regions (Figure 1).

**Figure 3:**
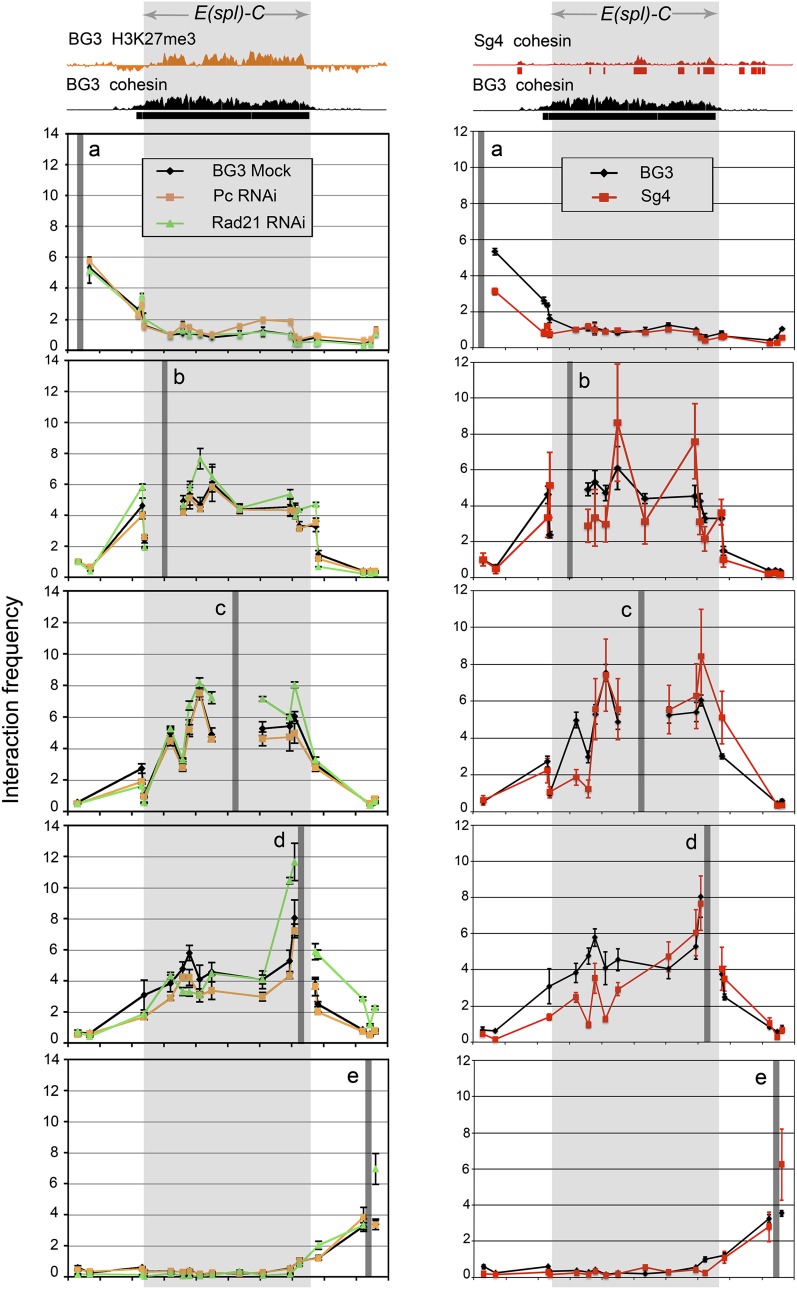
The E(spl)-C has a highly self-interactive structure independent of cohesin and PRC1 in BG3 and Sg4 cells. Chromosome conformation capture (3C) was performed as previously described (Schaaf *et al.* 2013b) using five anchors indicated by gray vertical bars. The precise anchor locations are shown in Figure 1. The shaded area indicates the extent of the E(spl)-C. The Y-axis gives the enrichment of 3C ligation to the anchor site relative to religated BAC DNA control. The left panels show the 3C enrichment for BG3 cells, and BG3 cells depleted for Rad21 for 4−5 d, and BG3 cells depleted for Pc for 4−6 d. At least two independent 3C libraries were made for each sample. Examples of Rad21 and Pc protein depletion are shown in Figure S2. Standard errors were calculated using all biological and qPCR replicates. The right panels compare the control BG3 analysis shown in the left panels to 3C analysis in Sg4 cells.

We used chromosome conformation capture (3C) analysis (Dekker *et al.* 2002) with five anchor *Eco*RI restriction sites over a 100-kb region to examine the looping structure of the E(spl)-C. Two anchors (a, e) are outside of the complex on either side, two (b, d) are at the inside ends, and one (c) is in the center of the complex (Figure 1; Figure 3). The interactions between these sites were determined by qPCR and normalized to a religated BAC control template. Anchors within the complex (b, c, d) exhibit high interaction frequencies with all sites tested within the complex, but little interaction with sites outside the complex (Figure 3). In contrast, anchors outside of the complex (a, e) exhibit local interactions with neighboring sites, but little interaction with sites within the E(spl)-C.

To test whether cohesin or PRC1 proteins facilitate the extensive looping interactions within the E(spl)-C, we performed 3C analysis after depletion of Rad21 or Pc. Although these depletions greatly increased transcription, there were only minor quantitative changes in the interaction of anchor d with immediately flanking regions (Figure 3, left panels). However, the overall pattern of extensive interactions within the complex, and little interaction outside of the complex, was not appreciably altered. This argues that the highly self-interactive structure of the E(spl)-C is not formed by cohesin or PRC1.

We considered the possibility that only low levels of cohesin and PRC1 are needed to form the structure and that RNAi depletion, which reduces cohesin or Pc levels by some 80%, is insufficient to alter the interactions. We thus examined the 3C structure of the E(spl)-C in Sg4 cells, in which there is no H3K27me3 in the E(spl)-C (Schaaf *et al.* 2009). Sg4 cells are derived from S2 cells, which do not express the Notch receptor. A few E(spl)-C genes (*HLHmδ*, *HLHmβ*, *HLHm3*, *HLHm6*) bind cohesin and Pol II in Sg4 cells and show low levels of expression that is not altered by cohesin depletion (Schaaf *et al.* 2009). Strikingly, the E(spl)-C has a 3C structure in Sg4 cells similar to that seen in BG3 cells, with only minor differences (Figure 3, right panels). We conclude, therefore, that the E(spl)-C self-interactive domain does not depend on cohesin or PRC1. Because this interactive domain is co-extensive with the cohesin-H2K27me3 region in BG3 cells, we hypothesize that this higher order structure establishes where cohesin and the PcG complexes bind and function when they are recruited to the E(spl)-C by unknown factors.

An early origin of DNA replication is centered over the E(spl)-C in BG3 and other cell lines (Figure 1; modENCODE Consortium *et al.* 2010). We thus also tested the possibility that the self-interactive structure in the E(spl)-C complex is the replication bubble present in early S-phase in the asynchronous cells used for 3C analysis. We blocked BG3 cells at the G1/S boundary and in G2 of the cell cycle using aphidicolin and dimethyl sulfoxide. There was no appreciable difference in the 3C structure of the E(spl)-C in the G1/S and G2 cells from that seen in the asynchronous cell population, indicating that the self-interactive structure is not the replication bubble present in S phase (Figure S3).

### Regulation of the E(spl)-C by the Chromator-Putzig/Z4 protein complex

We considered the idea that the E(spl)-C 3C structure may be formed by insulator proteins, which show less variation in binding between different cell types. However, inspection of genomic insulator ChIP data (Schwartz *et al.* 2012) revealed that there are no known insulator sites flanking or within the E(spl)-C in BG3 cells. Moreover, depletion of CP190, a protein required for the function of all known Drosophila insulators, does not significantly alter E(spl)-C expression in BG3 cells (Schaaf *et al.* 2009).

The Chromator (Chro) chromodomain protein binds at the telomere-proximal (right) end of the E(spl)-C in BG3 cells, and several kilobases upstream of the centromere-proximal end (Figure 1, modENCODE Consortium *et al.* 2010). Chro interacts with the Putzig (Pzg/Z4) zinc finger protein, and they colocalize on salivary gland polytene chromosomes (Gortchakov *et al.* 2005). Lack of Chro or Pzg/Z4 alters the structure of salivary gland polytene chromosomes, suggesting that they may play key roles in higher order chromatin architecture (Eggert *et al.* 2004; Rath *et al.* 2006). Pzg/Z4 also positively regulates Notch signaling in wing imaginal discs (Kugler and Nagel 2007). We thus tested the possibilities that Chro and Pzg/Z4 may participate in formation of the E(spl)-C 3C structure and regulate E(spl)-C transcription.

**Figure 4:**
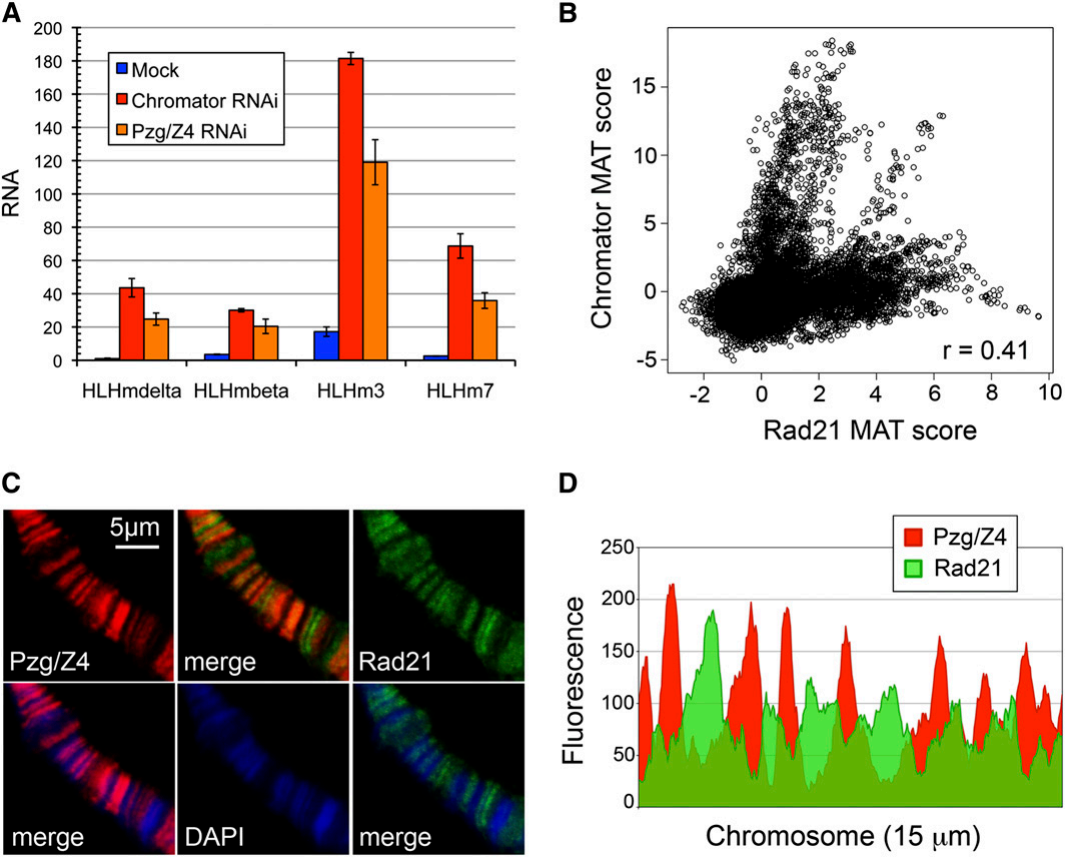
The Chromator-Pzg/Z4 protein complex regulates E(spl)-C expression in BG3 cells. (A) Chro and Pzg/Z4 RNAi treatment for 6 days increases expression of E(spl)-C genes. Examples of Pzg/Z4 protein depletion are shown in Figure S5. RNA quantification was performed as described in Figure 2. The results shown are from one experiment, and similar results were obtained in multiple independent experiments. (B) Plot of Rad21 *vs.* Chro ChIP-chip MAT scores enrichment over an example region and genome-wide Pearson correlation coefficient. (C) Representative example of salivary gland immunostaining for Rad21 and Pzg/Z4. (D) Plot of fluorescence intensity of Rad21 and Pzg/Z4 immunostaining over a representative 15-μm length of salivary gland polytene chromosome.

Depletion of Chro or Pzg/Z4 in BG3 cells increased expression of multiple genes in the E(spl)-C several-fold (Figure 4A). This includes a 10-fold or greater increase in expression of the *HLHm3* gene in the middle of the complex, even though the Chro-Pzg/Z4 binding sites are located outside the complex. The location of the Chro binding sites and the changes in E(spl)-C expression upon Chro or Pzg/Z4 depletion suggested that Chro and Pzg/Z4 might affect the E(spl)-C architecture, but depletion of Chro or Pzg/Z4 did not measurably alter the interactions measured by 3C (Figure S4).

Pzg/Z4 RNAi treatment decreased Pzg/Z4 protein and RNA in a dosage and time-dependent manner (Figure S5, A and B). We were unable to detect Chro protein by Western blot, and Chro RNAi treatment slightly increased Chro RNA transcripts (Figure S5A). Intriguingly, however, Chro dsRNA decreased Pzg/Z4 RNA to a similar extent as Pzg/Z4 RNAi treatment (Figure S5A), suggesting that Chro RNAi likely decreases the levels of both proteins.

We tested the possibility that Chro and Pzg/Z4 depletion might alter E(spl)-C expression indirectly by altering cohesin or Notch activity. Chro and Pzg/Z4 depletion increased *Rad21* and *Nipped-B* RNA levels, which would be expected to decrease E(spl)-C expression (Figure S5C). Although Rad21 RNA transcripts increased, the Rad21 protein levels were not appreciably altered (Figure S5B). We found, however, that the levels of the *Delta* and *Serrate* Notch ligand gene RNAs increased 2- to fourfold upon Chro or Pzg/Z4 RNAi treatment, suggesting that increased Notch receptor activation may contribute to increased E(spl)-C expression (Figure S5D). Chro binds to the *Delta* gene promoter region, but not to *Serrate* in BG3 cells (modENCODE Consortium *et al.* 2010).

We also considered the possibility the Chro-Pzg/Z4 complex might control E(spl)-C transcription by influencing cohesin binding based on immunostaining of salivary gland polytene chromosomes and genomic ChIP patterns. Chro, Pzg/Z4, and cohesin all bind preferentially to polytene interband regions, which represent less compacted, transcriptionally active chromatin, as revealed by DAPI staining (Figure 4, B and C). In most regions, the peaks of Chro (not shown) and Pzg/Z4 staining interdigitate with the cohesin peaks (Figure 4D). Similarly, when genomic ChIP enrichment for Chro and Rad21 in BG3 cells are plotted against each other, regions with high Rad21 usually have low Chro binding, and *vice versa* (Figure 4B). This also reveals, however, that there are sites where cohesin and Chro binding overlap, and that there are often low levels of cohesin at Chro-binding sites and *vice versa*. These overlaps give rise to a significant genome-wide correlation of 0.41 between Chro and Rad21 binding. We tested the possibility that Chro and Pzg/Z4 could form boundaries that limit where cohesin binds, and influence cohesin binding to the E(spl)-C by performing Rad21 ChIP after Pzg/Z4 depletion. We found, however, that Pzg/Z4 depletion did not alter cohesin levels at the *HLHmβ* and *HLHm3* promoter regions (Figure S5E). We posit, therefore, that the effect of Chro and Pzg/Z4 on E(spl)-C expression is most likely caused by increased expression of Notch ligands.

## Discussion

**Figure 5:**
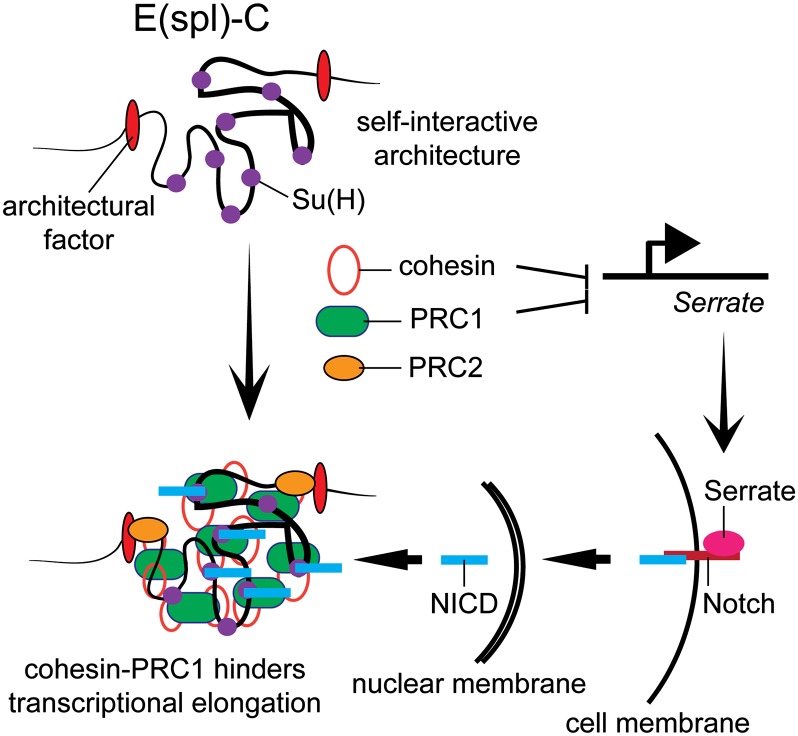
Architecture and regulation of the E(spl)-C in BG3 cells. We theorize that unknown architectural proteins (red ovals) form the self-interactive domain that encompasses the E(spl)-C, and that this domain dictates where cohesin (red ring) and the PRC1 (green oval) and PRC2 (orange oval) Polycomb group complexes bind when they are recruited by as yet unidentified factors. PRC2 is usually found only at the ends of H3K27me3 domains (Schwartz *et al.* 2010). Cohesin and PRC1 indirectly control E(spl)-C transcription by repressing expression of the *Serrate* gene that encodes a ligand (pink oval) for the Notch transmembrane receptor. The Chro-Pzg/Z4 protein complex similarly inhibits *Serrate* expression. Notch activation releases the NICD activator fragment (light blue rectangle) that translocates to the nucleus and binds to Su(H) (purple circles). NICD is not detected at all genes in the complex, even some that are active in a Notch-dependent manner. We propose that cohesin and PRC1 also directly hinder transition of paused RNA Pol II to elongation at the active E(spl)-C genes, as they do at several other genes (Fay *et al.* 2011; Schaaf *et al.* 2013a; Schaaf *et al.* 2013b).

In these studies we investigated the regulation of the E(spl)-C complex by cohesin, PRC1, and the Chro-Z4/Pzg protein complex in BG3 cells, in which the E(spl)-C has a rare restrained state with a cohesin-H3K27me3 overlap. We find that the E(spl)-C has a highly self-interactive structure that is unexpectedly independent of these protein complexes and the level of gene expression. Depletion of any of these three protein complexes, however, significantly increases E(spl)-C transcription. As discussed herein, the effects of these three protein complexes on E(spl)-C expression likely reflect changes in expression of Notch ligands, and in the cases of cohesin and PRC1, potentially direct effects on activator and Pol II activity at the E(spl)-C genes (Figure 5).

### Architecture of the E(spl)-C domain

3C analysis revealed that the E(spl)-C has a structure in which all positions within the complex interact with each other at a high frequency, but not with flanking regions. Surprisingly, we found that this architecture is independent of cohesin, the PcG complexes, the Chro-Pzg/Z4 complex, transcription, and stage of the cell cycle. We thus do not know the factors that establish this striking architecture, which defines the E(spl)-C as a structurally independent domain. We also do not yet know the factors that control recruitment of cohesin and PcG complexes to the locus. We speculate, however, that this architecture coordinates transcriptional control of the entire E(spl)-C, based on the finding that in BG3 cells, cohesin, PRC1, and the H2Aub and H3K27me3 histone modifications made by the PRC1 and PRC2 complexes are co-extensive within this architectural domain. Although no known insulators or boundary elements flank the E(spl)-C, and depletion of the CP190 protein required for activity of all known Drosophila insulators does not alter E(spl)-C expression, it is likely that the unknown factors that form this structure limit the spread of these protein complexes and histone marks. The E(spl)-C architectural domain may be evolutionarily significant, because Notch-regulated *Enhancer of split* complexes with similar structures are conserved in insects and crustaceans over 420 million years (Maeder *et al.* 2007; Duncan and Dearden 2010).

Possible clues to the identities of the factors that control the E(spl)-C architecture and/or the recruitment of cohesin and PcG complexes may arise in genetic screens for factors that alter E(spl)-C sensitive phenotypes, such as the *N^spl-1^* rough eye and bristle phenotypes. These phenotypes are sensitive to mutations in the E(spl)-C and cohesin genes in a highly dosage-sensitive manner, and modest changes in the E(spl)-C architecture or recruitment of cohesin or PcG proteins may have similar effects (Nagel and Preiss 1999; Rollins *et al.* 1999; Schaaf *et al.* 2009).

There is coordinate regulation of gene complexes by cohesin in mammalian cells. The *Protocadherin beta* (*Pchdb*) gene complex is down-regulated in the embryonic fibroblasts and brains of mice heterozygous mutant for the Nipbl cohesin loading factor, and brains of mice that are homozygous mutant for the SA1 cohesin subunit (Kawauchi *et al.* 2009; Remeseiro *et al.* 2012), and cohesin is involved in enhancer-promoter looping in the *Protocadherin alpha* (*Pchda*) complex, helping determine which genes in the complex are active (Guo *et al.* 2012; Monahan *et al.* 2012). Although this is a positive role for cohesin, as opposed to the repressive role that occurs in the E(spl)-C, it is possible that the protocadherin gene clusters also have a higher-order architecture that dictates how cohesin functions within the gene complex. Recent genome-wide analysis also indicates that there are constitutive higher order looping architectures that may organize cell-type specific interactions on a shorter scale, and that cohesin contributes to both types of structures (Phillips-Cremins *et al.* 2013).

### Effects of cohesin, PRC1, and Chro-Pzg/Z4 on Notch signaling

Previous studies showed that depletion of cohesin or PRC1 increases expression of the *Serrate* Notch ligand gene (Schaaf *et al.* 2009, 2013b). This likely explains part of the increase in E(spl)-C transcription upon cohesin and PRC1 depletion, because the E(spl)-C genes are directly activated by Notch. Consistent with this idea, we detected increases in NICD association with the *HLHmβ* and *HLHm3* genes upon cohesin or PRC1 depletion. EDTA treatment confirms that increasing Notch activation increases NICD binding to the E(spl)-C genes.

Because cohesin and PRC1, unlike the Chro-Pzg/Z4 complex, bind directly to the E(spl)-C, it is also possible that they also directly control association of NICD with the Su(H) protein bound upstream of the active genes. For example, they could potentially interact with NICD or the Su(H) complex, and interfere with NICD association, or somehow facilitate ubiquitination and degradation of NICD. The lack of an effect of cohesin or PRC1 depletion on NICD association with E(spl)-C genes after EDTA treatment does not rule out this possibility, because under these conditions, the amount of NICD is no longer limiting.

It remains to be determined whether the multiple effects of cohesin on Notch function seen in Drosophila, including regulation of Notch ligand and target genes, also occur in mammals. If so, this could underlie many of the development deficits seen in Cornelia de Lange syndrome, caused by mutations in *NIPBL* and cohesin subunit genes (Liu and Krantz 2009). Mutations in Notch receptor and ligand genes cause Alagille and other syndromes that affect many of the same tissues as Cornelia de Lange syndrome (Penton *et al.* 2012).

### Other potential direct roles of cohesin and PRC1 in E(spl)-C repression

We also cannot rule out the possibility that cohesin and PRC1 directly repress E(spl)-C transcription independently of any effects on Notch ligand expression or NICD association with the E(spl)-C genes. This is because both bind throughout the complex, and the PRC1-generated H2Aub repressive histone mark is co-extensive with the E(spl)-C architectural domain. Importantly, all genes in BG3 cells that show rare extended overlap of cohesin and the PRC2-generated H3K27me3 modification, such as the *invected* and *engrailed* gene complex, show substantial increases in transcription upon cohesin or PRC1 depletion, even though they are not Notch activated (Fay *et al.* 2011; Schaaf *et al.* 2009, 2013a,b). It is highly unlikely that cohesin or PRC1 depletion increases the expression of all the diverse activators that control these genes, and more likely that cohesin and PRC1 directly repress their transcription.

At all genes examined that are strongly repressed by cohesin, cohesin restricts the transition of paused RNA Pol II into elongation, irrespective of whether or not they have the H3K27me3 mark (Fay *et al.* 2011). PRC1 restricts entry of paused Pol II into elongation at active genes that bind cohesin and PRC1, but lack PRC2 and the H3K27me3 modification (Schaaf *et al.* 2013b). We thus posit that cohesin and PRC1 together restrict transition of the paused Pol II present at the active E(spl)-C genes into elongation. Because codepletion of cohesin and PRC1 does not synergistically increase transcription, we think it is likely that they function together at the same step. Cohesin and PRC1 directly interact with each other, and cohesin facilitates binding of PRC1 to active genes that lack the H3K27me3 mark (Strübbe *et al.* 2011; Schaaf *et al.* 2013b). Cohesin depletion, however, does not significantly alter PRC1 association with the E(spl)-C, likely because PRC1 binding is stabilized by the known interaction of PRC1 with H2K27me3 (Fischle *et al.* 2003). PRC1 is thus not sufficient to repress E(spl)-C transcription in the absence of cohesin, indicating that cohesin has roles that extend beyond its interaction with PRC1.

## Supplementary Material

Supporting Information

## References

[bib1] Bailey A. M. Posakony J. W., 1995 Suppressor of hairless directly activates transcription of enhancer of split complex genes in response to Notch receptor activity. Genes Dev. 9: 2609–2622 7590239 10.1101/gad.9.21.2609

[bib2] Chien R. Zeng W. Ball A. R. Yokomori K., 2011 a Cohesin: a critical chromatin organizer in mammalian gene regulation. Biochem. Cell. Biol. 89: 445–458 21851156 10.1139/o11-039 PMC4056987

[bib3] Chien R. Zeng W. Kawauchi S. Bender M. A. Santos R., 2011 b Cohesin mediates chromatin interactions that regulate mammalian β-globin expression. J. Biol. Chem. 286: 17870–17878 21454523 10.1074/jbc.M110.207365 PMC3093862

[bib4] Dekker J. Rippe K. Dekker M. Kleckner N., 2002 Capturing chromosome conformation. Science 295: 1306–1311 11847345 10.1126/science.1067799

[bib5] Delest A. Sexton T. Cavalli G., 2012 Polycomb: a paradigm for genome organization from one to three dimensions. Curr. Opin. Cell Biol. 24: 405–414 22336329 10.1016/j.ceb.2012.01.008

[bib6] Dorsett D., 2011 Cohesin: genomic insights into controlling gene transcription and development. Curr. Opin. Genet. Dev. 21: 199–206 21324671 10.1016/j.gde.2011.01.018 PMC3070859

[bib7] Dorsett D. Merkenschlager M., 2013 Cohesin at active genes: a unifying theme for cohesin and gene expression from model organisms to humans. Curr. Opin. Cell Biol. 25: 327–333 23465542 10.1016/j.ceb.2013.02.003 PMC3691354

[bib8] Dorsett D. Ström L., 2012 The ancient and evolving roles of cohesin in gene expression and DNA repair. Curr. Biol. 22: R240–R250 22497943 10.1016/j.cub.2012.02.046 PMC3327610

[bib9] Dorsett D. Eissenberg J. C. Misulovin Z. Martens A. Redding B., 2005 Effects of sister chromatid cohesion proteins on *cut* gene expression during wing development in Drosophila. Development 132: 4743–4753 16207752 10.1242/dev.02064 PMC1635493

[bib10] Duncan E. J. Dearden P. K., 2010 Evolution of a genomic regulatory domain: the role of gene co-option and gene duplication in the *Enhancer of split* complex. Genome Res. 20: 917–928 20458100 10.1101/gr.104794.109 PMC2892093

[bib11] Eggert H. Gortchakov A. Saumweber H., 2004 Identification of the Drosophila interband-specific protein Z4 as a DNA-binding zinc-finger protein determining chromosomal structure. J. Cell Sci. 117: 4253–4264 15292401 10.1242/jcs.01292

[bib12] Faure A. J. Schmidt D. Watt S. Schwalie P. C. Wilson M. D., 2012 Cohesin regulates tissue-specific expression by stabilizing highly occupied cis-regulatory modules. Genome Res. 22: 2163–2175 22780989 10.1101/gr.136507.111 PMC3483546

[bib13] Fay A. Misulovin Z. Li J. Schaaf C. A. Gause M., 2011 Cohesin selectively binds and regulates genes with paused RNA polymerase. Curr. Biol. 21: 1624–1634 21962715 10.1016/j.cub.2011.08.036 PMC3193539

[bib14] Fischle W. Wang Y. Jacobs S. A. Kim Y. Allis C. D., 2003 Molecular basis for the discrimination of repressive methyl-lysine marks in histone H3 by Polycomb and HP1 chromodomains. Genes Dev. 17: 1870–1881 12897054 10.1101/gad.1110503 PMC196235

[bib15] Fryer C. J. Lamar E. Turbachova I. Kintner C. Jones K. A., 2002 Mastermind mediates chromatin-specific transcription and turnover of the Notch enhancer complex. Genes Dev. 16: 1397–1411 12050117 10.1101/gad.991602 PMC186317

[bib16] Fryer C. J. White J. B. Jones K. A., 2004 Mastermind recruits CycC:CDK8 to phosphorylate the Notch ICD and coordinate activation with turnover. Mol. Cell 16: 509–520 15546612 10.1016/j.molcel.2004.10.014

[bib17] Gause M. Webber H. A. Misulovin Z. Haller G. Rollins R. A., 2008 Functional links between Drosophila Nipped-B and cohesin in somatic and meiotic cells. Chromosoma 117: 51–66 17909832 10.1007/s00412-007-0125-5 PMC2258212

[bib18] Gortchakov A. A. Eggert H. Gan M. Mattow J. Zhimulev I. F., 2005 Chriz, a chromodomain protein specific for the interbands of *Drosophila melanogaster* polytene chromosomes. Chromosoma 114: 54–66 15821938 10.1007/s00412-005-0339-3

[bib19] Guo Y. Monahan K. Wu H. Gertz J. Varley K. E., 2012 CTCF/cohesin-mediated DNA looping is required for protocadherin α promoter choice. Proc. Natl. Acad. Sci. USA 109: 21081–21086 23204437 10.1073/pnas.1219280110 PMC3529044

[bib20] Horsfield J. A. Print C. G. Mönnich M., 2012 Diverse developmental disorders from the one ring: distinct molecular pathways underlie the cohesinopathies. Front. Genet. 3: 171 22988450 10.3389/fgene.2012.00171 PMC3439829

[bib21] Johnson W. E. Li W. Meyer C. A. Gottardo R. Carroll J. S., 2006 Model-based analysis of tiling-arrays for ChIP-chip. Proc. Natl. Acad. Sci. USA 103: 12457–12462 16895995 10.1073/pnas.0601180103 PMC1567901

[bib22] Kagey M. H. Newman J. J. Bilodeau S. Zhan Y. Orlando D. A., 2010 Mediator and cohesin connect gene expression and chromatin architecture. Nature 467: 430–435 20720539 10.1038/nature09380 PMC2953795

[bib23] Kassis J. A. Brown J. L., 2013 Polycomb group response elements in Drosophila and vertebrates. Adv. Genet. 81: 83–118 23419717 10.1016/B978-0-12-407677-8.00003-8 PMC4157523

[bib24] Kawauchi S. Calof A. L. Santos R. Lopez-Burks M. E. Young C. M., 2009 Multiple organ system defects and transcriptional dysregulation in the *Nipbl*(+/−) mouse, a model of Cornelia de Lange Syndrome. PLoS Genet. 5: e1000650 19763162 10.1371/journal.pgen.1000650 PMC2730539

[bib25] Krejcí A. Bray S., 2007 Notch activation stimulates transient and selective binding of Su(H)/CSL to target enhancers. Genes Dev. 21: 1322–1327 17545467 10.1101/gad.424607 PMC1877745

[bib26] Kugler S. J. Nagel A. C., 2007 putzig is required for cell proliferation and regulates notch activity in Drosophila. Mol. Biol. Cell 18: 3733–3740 17634285 10.1091/mbc.E07-03-0263 PMC1995712

[bib27] Lin C. Y. Lovén J. Rahl P. B. Paranal R. M. Burge C. B., 2012 Transcriptional amplification in tumor cells with elevated c-Myc. Cell 151: 56–67 23021215 10.1016/j.cell.2012.08.026 PMC3462372

[bib28] Liu J. Krantz I. D., 2009 Cornelia de Lange syndrome, cohesin, and beyond. Clin. Genet. 76: 303–314 19793304 10.1111/j.1399-0004.2009.01271.x PMC2853897

[bib29] Maeder M. L. Polansky B. J. Robson B. E. Eastman D. A., 2007 Phylogenetic footprinting analysis in the upstream regulatory regions of the Drosophila *enhancer of split* genes. Genetics 177: 1377–1394 18039873 10.1534/genetics.107.070425 PMC2147979

[bib30] Mannini L. Menga S. Musio A., 2010 The expanding universe of cohesin functions: a new genome stability caretaker involved in human disease and cancer. Hum. Mutat. 31: 623–630 20513141 10.1002/humu.21252

[bib31] Merkenschlager M. Odom D. T., 2013 CTCF and cohesin: linking gene regulatory elements with their targets. Cell 152: 1285–1297 23498937 10.1016/j.cell.2013.02.029

[bib32] Misulovin Z. Schwartz Y. B. Li X. Y. Kahn T. G. Gause M., 2008 Association of cohesin and Nipped-B with transcriptionally active regions of the *Drosophila melanogaster* genome. Chromosoma 117: 89–102 17965872 10.1007/s00412-007-0129-1 PMC2258211

[bib33] modENCODE Consortium Roy S. Ernst J. Kharchenko P. V. Kheradpour P., 2010 Identification of functional elements and regulatory circuits by Drosophila modENCODE. Science 330: 1787–1797 21177974 10.1126/science.1198374 PMC3192495

[bib34] Monahan K. Rudnick N. D. Kehayova P. D. Pauli F. Newberry K. M., 2012 Role of CCCTC binding factor (CTCF) and cohesin in the generation of single-cell diversity of protocadherin-α gene expression. Proc. Natl. Acad. Sci. USA 109: 9125–9130 22550178 10.1073/pnas.1205074109 PMC3384188

[bib35] Nagel A. C. Preiss A., 1999 *Notch^spl^* is deficient for inductive processes in the eye, and *E(spl)^D^* enhances split by interfering with proneural activity. Dev. Biol. 208: 406–415 10191054 10.1006/dbio.1999.9203

[bib36] Nasmyth K., 2011 Cohesin: a catenase with separate entry and exit gates? Nat. Cell Biol. 13: 1170–1177 21968990 10.1038/ncb2349

[bib37] Nie Z. Hu G. Wei G. Cui K. Yamane A., 2012 c-Myc is a universal amplifier of expressed genes in lymphocytes and embryonic stem cells. Cell 151: 68–79 23021216 10.1016/j.cell.2012.08.033 PMC3471363

[bib38] Penton A. L. Leonard L. D. Spinner N. B., 2012 Notch signaling in human development and disease. Semin. Cell Dev. Biol. 23: 450–457 22306179 10.1016/j.semcdb.2012.01.010 PMC3638987

[bib39] Phillips-Cremins J. E. Sauria M. E. Sanyal A. Gerasimova T. I. Lajoie B. R., 2013 Architectural protein subclasses shape 3D organization of genomes during lineage commitment. Cell 153: 1281–1295 23706625 10.1016/j.cell.2013.04.053 PMC3712340

[bib40] Rath U. Ding Y. Deng H. Qi H. Bao X., 2006 The chromodomain protein, Chromator, interacts with JIL-1 kinase and regulates the structure of Drosophila polytene chromosomes. J. Cell Sci. 119: 2332–2341 16723739 10.1242/jcs.02960

[bib41] Remeseiro S. Losada A., 2013 Cohesin, a chromatin engagement ring. Curr. Opin. Cell Biol. 25: 63–71 23219370 10.1016/j.ceb.2012.10.013

[bib42] Remeseiro S. Cuadrado A. Gómez-López G. Pisano D. G. Losada A., 2012 A unique role of cohesin-SA1 in gene regulation and development. EMBO J. 31: 2090–2102 22415368 10.1038/emboj.2012.60 PMC3343463

[bib43] Rhodes J. M. Bentley F. K. Print C. G. Dorsett D. Misulovin Z., 2010 Positive regulation of c-Myc by cohesin is direct, and evolutionarily conserved. Dev. Biol. 344: 637–649 20553708 10.1016/j.ydbio.2010.05.493 PMC2941799

[bib44] Rollins R. A. Morcillo P. Dorsett D., 1999 Nipped-B, a Drosophila homologue of chromosomal adherins, participates in activation by remote enhancers in the *cut* and *Ultrabithorax* genes. Genetics 152: 577–593 10353901 10.1093/genetics/152.2.577 PMC1460629

[bib45] Schaaf C. A. Misulovin Z. Sahota G. Siddiqui A. M. Schwartz Y. B., 2009 Regulation of the Drosophila *Enhancer of split* and *invected-engrailed* gene complexes by sister chromatid cohesion proteins. PLoS ONE 4: e6202 19587787 10.1371/journal.pone.0006202 PMC2703808

[bib46] Schaaf C. A. Kwak H. Koenig A. Misulovin Z. Gohara D. W., 2013 a Genome-wide control of RNA polymerase II activity by cohesin. PLoS Genet. 9: e1003382 23555293 10.1371/journal.pgen.1003382 PMC3605059

[bib47] Schaaf C. A. Misulovin Z. Gause M. Koenig A. Gohara D. W., 2013 b Cohesin and Polycomb proteins functionally interact to control transcription at silenced and active genes. PLoS Genet. 9: e1003560 23818863 10.1371/journal.pgen.1003560 PMC3688520

[bib51] Schwartz Y. B. Kahn T. G. Stenberg P. Ohno K. Bourgon R. Pirrotta V., 2010 Alternative epigenetic chromatin states of polycomb target genes. PLoS Genet. 6: e1000805 20062800 10.1371/journal.pgen.1000805 PMC2799325

[bib48] Schwartz Y. B. Linder-Basso D. Kharchenko P. V. Tolstorukov M. Y. Kim M., 2012 Nature and function of insulator protein binding sites in the Drosophila genome. Genome Res. 22: 2188–2198 22767387 10.1101/gr.138156.112 PMC3483548

[bib49] Seitan V. C. Hao B. Tachibana-Konwalski K. Lavagnolli T. Mira-Bontenbal H., 2011 A role for cohesin in T-cell-receptor rearrangement and thymocyte differentiation. Nature 476: 467–471 21832993 10.1038/nature10312 PMC3179485

[bib50] Strübbe G. Popp C. Schmidt A. Pauli A. Ringrose L., 2011 Polycomb purification by in vivo biotinylation tagging reveals cohesin and Trithorax group proteins as interaction partners. Proc. Natl. Acad. Sci. USA 108: 5572–5577 21415365 10.1073/pnas.1007916108 PMC3078387

