# Supplementary material for: The Drosophila Enhancer of split Gene Complex: Architecture and Coordinate Regulation by Notch, Cohesin, and Polycomb Group Proteins
Source: G3 (Bethesda). 2013 Oct 1;3(10):1785–94. doi: 10.1534/g3.113.007534 (PMC3789803; doi:10.1534/g3.113.007534)
Supplement: Supporting Information [file supp_3_10_1785__index.html]

The Drosophila Enhancer of split Gene Complex: Architecture and Coordinate Regulation by Notch, Cohesin, and Polycomb Group Proteins — Supporting Information 

# The Drosophila *Enhancer of split* Gene Complex: Architecture and Coordinate Regulation by Notch, Cohesin, and Polycomb Group Proteins

## Supporting Information for Schaaf *et al.*, 2013

**Files in this Data Supplement:**

- Supporting Information - Figures S1-S5 (PDF, 1 MB)
- Figure S1 - (A) Detailed maps of HLHmb and HLHm3 genes in BG3 cells. Tracks are as described in Figure 1. (B) Cohesin depletion does not alter histone H3 lysine 27 trimethylation (H3K27me3) at the E(spl)-C in BG3 cells. (PDF, 559 KB)
- Figure S2 - Simultaneous cohesin and PRC1 depletion does not synergistically increase E(spl)-C expression in BG3 cells. (PDF, 517 KB)
- Figure S3 - The higher order structure of the E(spl)-C is independent of the cell cycle stage in BG3 cells. (PDF, 641 KB)
- Figure S4 - The higher order structure of the E(spl)-C is independent of the Chromator-Pzg/Z4 protein complex in BG3 cells. (PDF, 437 KB)
- Figure S5 - Depletion of the Chromator-Pzg/Z4 complex increases cohesin and Notch ligand gene expression in BG3 cells. (PDF, 645 KB)
