# Supplementary material for: The Drosophila Enhancer of split Gene Complex: Architecture and Coordinate Regulation by Notch, Cohesin, and Polycomb Group Proteins
Source: G3 (Bethesda). 2013 Oct 1;3(10):1785–94. doi: 10.1534/g3.113.007534 (PMC3789803; doi:10.1534/g3.113.007534)
Supplement: Supporting Information [file supp_g3.113.007534_007534SI.pdf]

G3 · Genes | Genomes | Genetics

The *Drosophila Enhancer of split* gene complex: architecture and coordinate regulation by Notch, cohesin and Polycomb group proteins

Cheri A. Schaaf, Ziva Misulovin, Maria Gause, Amanda Koenig, and Dale Dorsett

Edward A Doisy Department of Biochemistry and Molecular Biology, Saint Louis University School of Medicine, Saint Louis, Missouri, USA

DOI: 10.1534/g3.113.007534

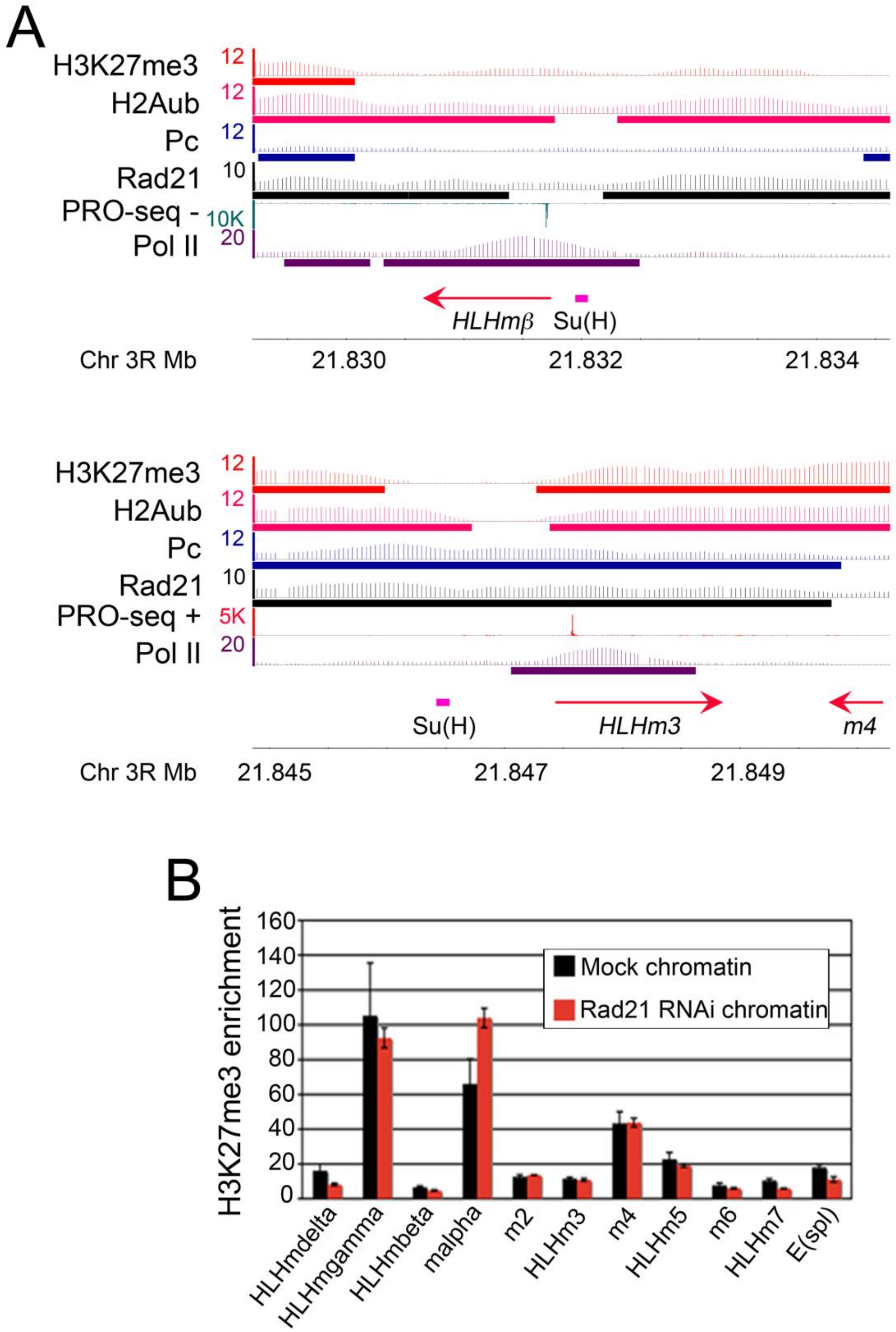

Figure S1 (A) Detailed maps of HLHmb and HLHm3 genes in BG3 cells. Tracks are as described in Figure 1. (B) Cohesin depletion does not alter histone H3 lysine 27 trimethylation (H3K27me3) at the E(spl)-C in BG3 cells. ChIP-PCR was performed for H3K27me3 at the promoters of several genes in the E(spl)-C in BG3 cells, and BG3 cells depleted for cohesin (Rad21) for five days. Enrichment was calculated relative to an empty site control on chromosome 3R.

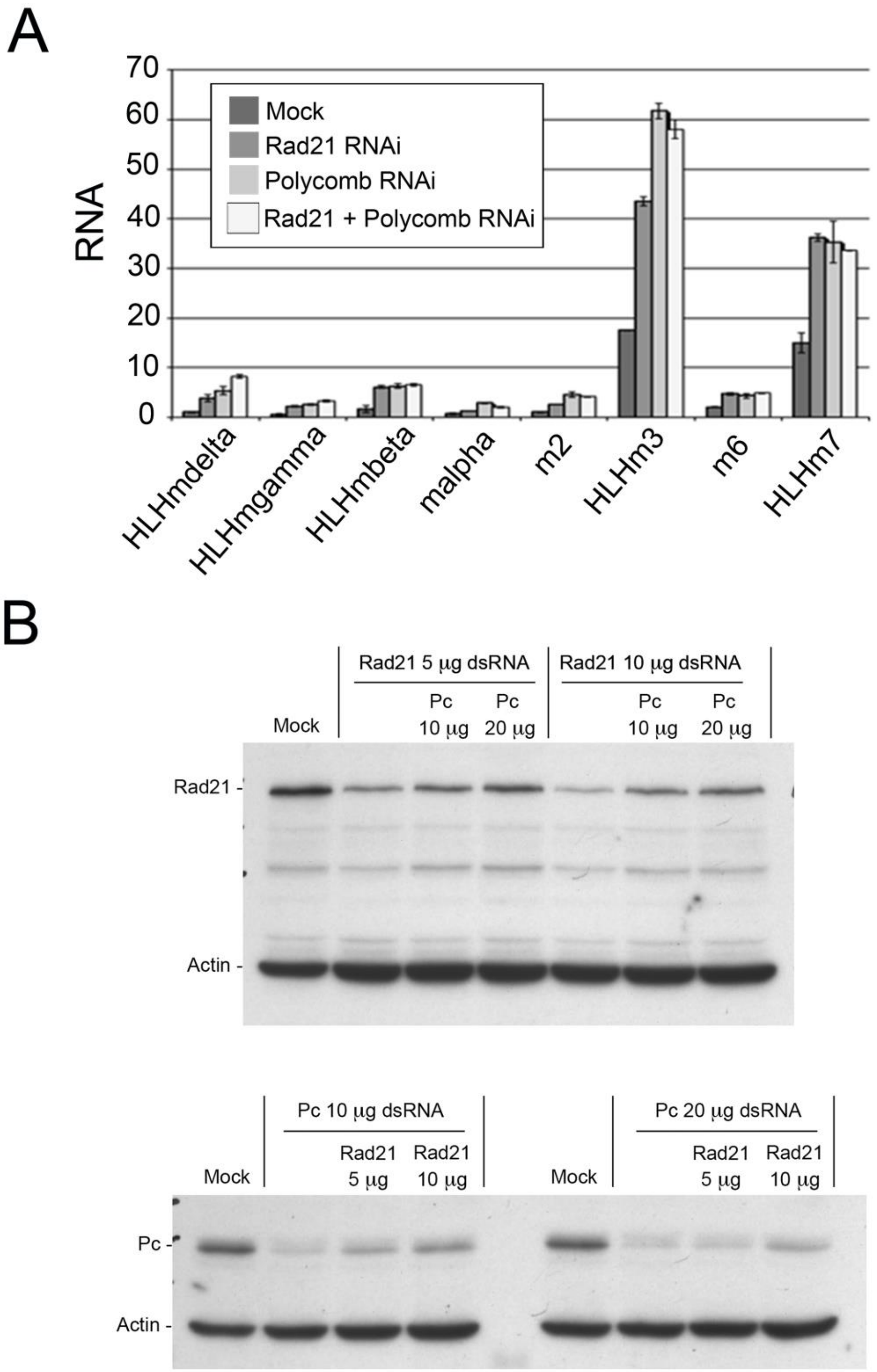

Figure S2 Simultaneous cohesin and PRC1 depletion does not synergistically increase E(spl)-C expression in BG3 cells. (A) E(spl)-C RNA levels in control BG3 cells, and BG3 cells depleted for Rad21, Pc, or both were measured by RT-qPCR as described in Figure 1. The BG3 cells were treated for five days with 5 μg of Rad21 dsRNA, 10 μg Pc dsRNA, or 5 μg of Rad21 plus 10 μg of Pc dsRNA per well. (B) The western blot shows extent of Rad21 and Pc protein depletion after five days of treatment with the indicated combinations of Rad21 and Pc dsRNA per well.

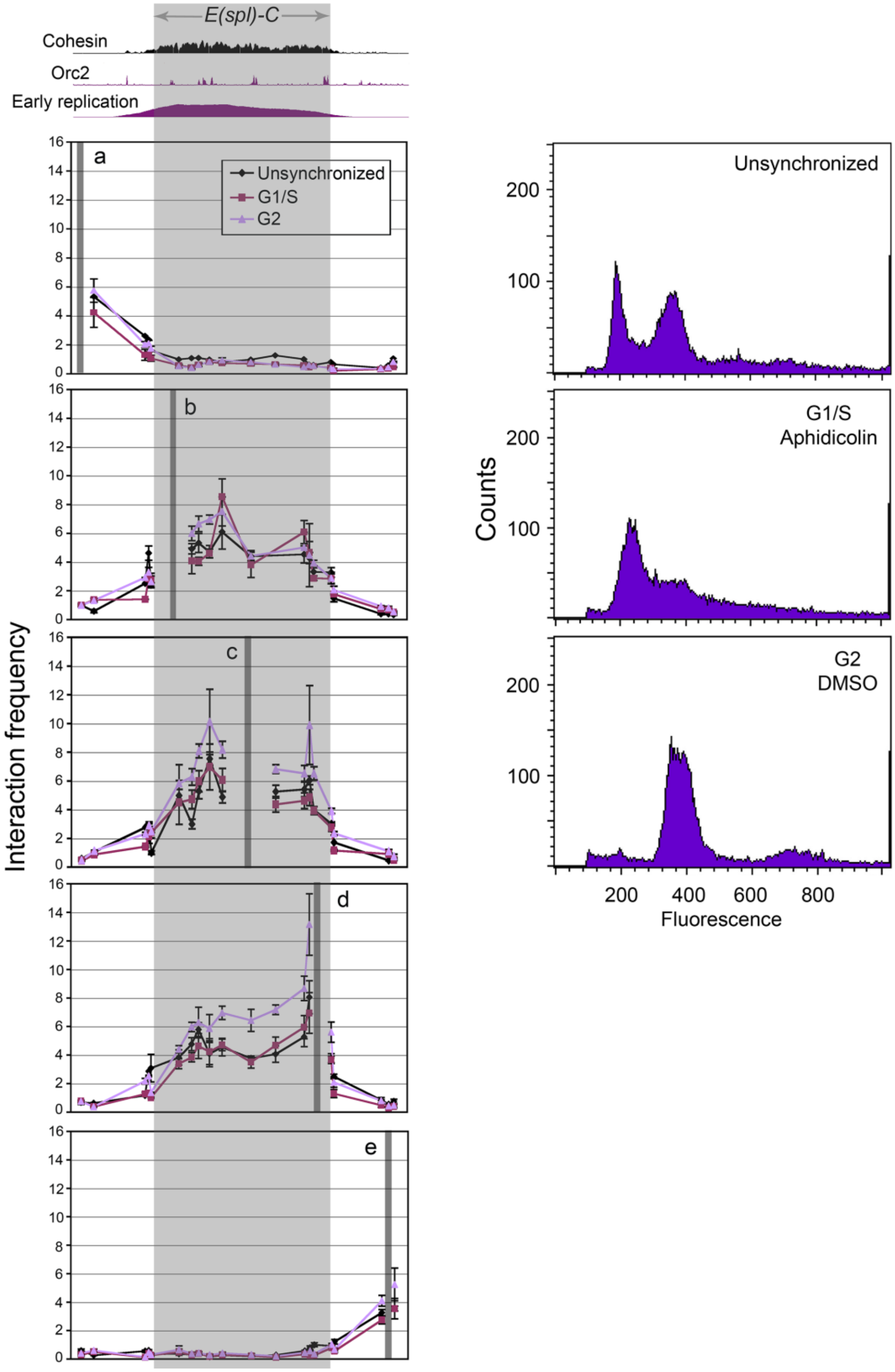

Figure S3 The higher order structure of the E(spl)-C is independent of the cell cycle stage in BG3 cells. The left panels compare the 3C analysis of control BG3 cells from Figure 1 to the 3C analysis of BG3 cells blocked at the G1/S boundary by treatment with 5 µg per mL aphidicolin for 26 hours, and BG3 cells blocked in G2 by treatment with 3% DMSO for 26 hours. The FACS analysis showing the cell cycle stages for each sample are shown in the panels on the right.

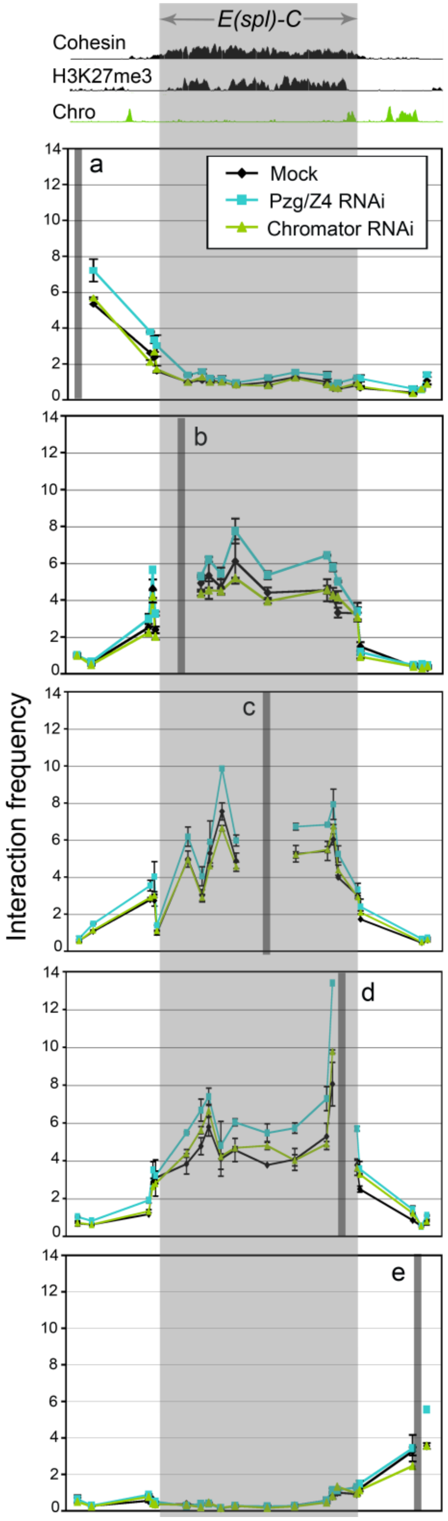

Figure S4 The higher order structure of the E(spl)-C is independent of the Chromator-Pzg/Z4 protein complex in BG3 cells. The panels compare the 3C analysis of control BG3 cells from Figure 1 to 3C analysis of BG3 cells treated with 40 µg of Pzg/Z4 or Chro dsRNA per well for six days. The Pzg/Z4 protein depletion is shown in Figure S5.

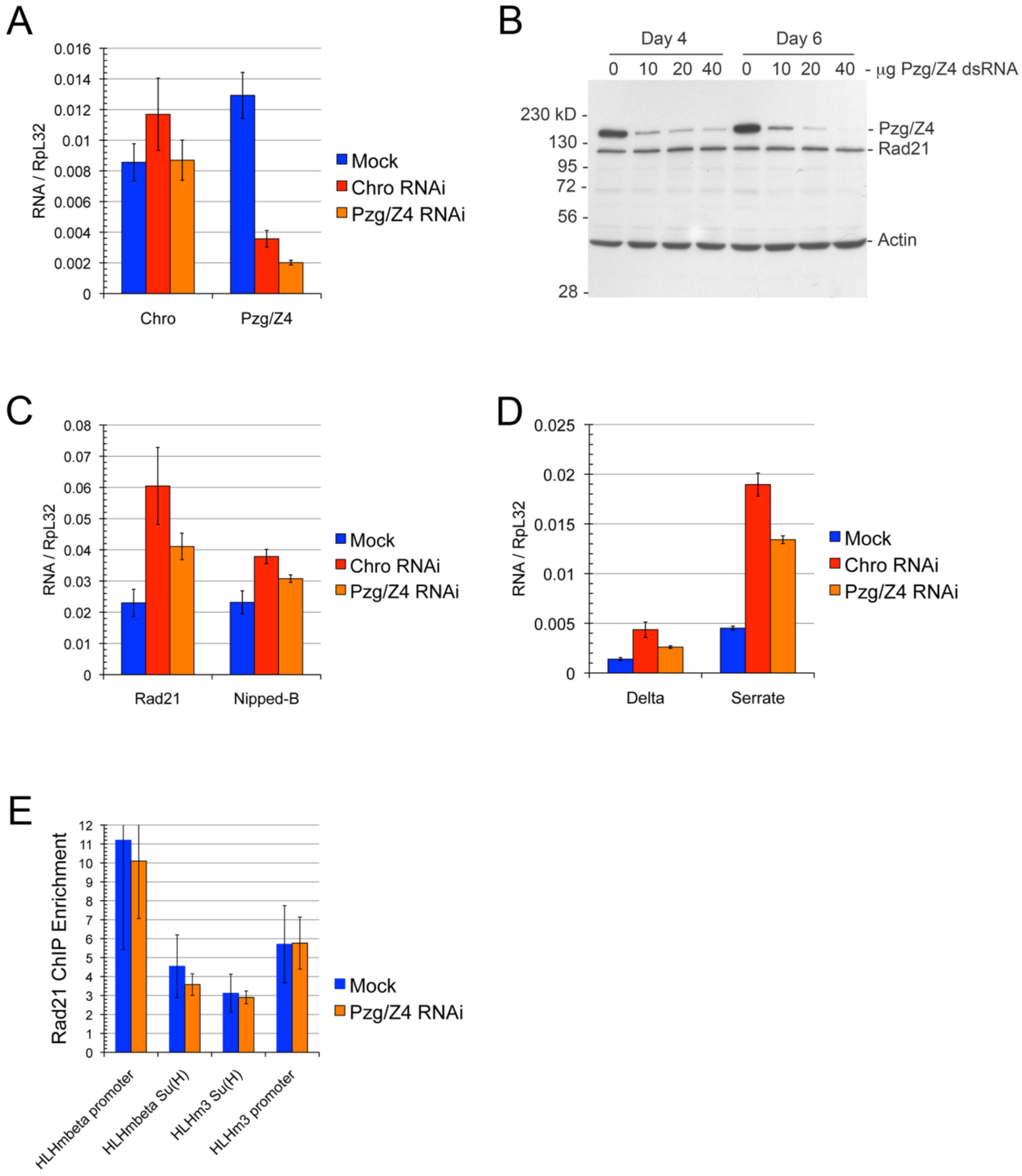

Figure S5 Depletion of the Chromator-Pzg/Z4 complex increases cohesin and Notch ligand gene expression in BG3 cells. (A) Chro and Pzg/Z4 RNAi treatment (40 µg dsRNA per well) for six days decreases Pzg/Z4 RNA levels. (B) The western blot shows the reduction of Pzg/Z4 protein levels with the indicated Pzg/Z4 dsRNA levels for the indicated time of treatment. (C) Chro and Pzg/Z4 RNAi treatment for six days increases *Rad21* and *Nipped-B* RNA levels. Panel B shows that Rad21 protein levels do not show a corresponding increase. (D) Chro and Pzg/Z4 RNAi treatment increase *Delta* and *Serrate* RNA transcripts. (E) Pzg/Z4 depletion does not alter cohesin (Rad21) binding to *HLHmb* and *HLHm3* genes in the E(spl)-C.
