# Supplementary material for: The Drosophila Enhancer of split Gene Complex: Architecture and Coordinate Regulation by Notch, Cohesin, and Polycomb Group Proteins
Source: G3 (Bethesda). 2013 Oct 1;3(10):1785–94. doi: 10.1534/g3.113.007534 (PMC3789803; doi:10.1534/g3.113.007534)
Supplement: Supporting Information [file supp_g3.113.007534_FigureS1.pdf]

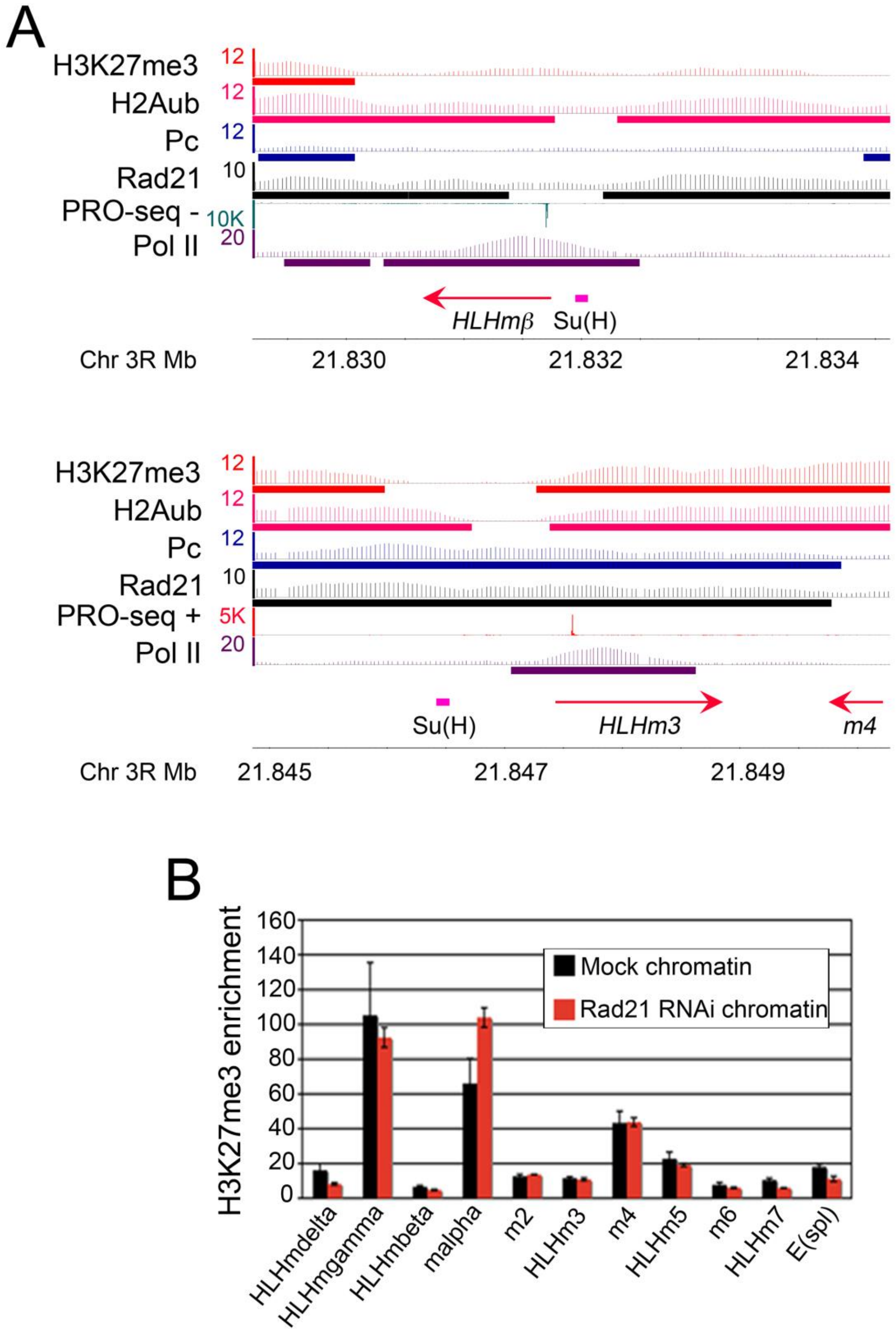

Figure S1 (A) Detailed maps of HLHmb and HLHm3 genes in BG3 cells. Tracks are as described in Figure 1. (B) Cohesin depletion does not alter histone H3 lysine 27 trimethylation (H3K27me3) at the E(spl)-C in BG3 cells. ChIP-PCR was performed for H3K27me3 at the promoters of several genes in the E(spl)-C in BG3 cells, and BG3 cells depleted for cohesin (Rad21) for five days. Enrichment was calculated relative to an empty site control on chromosome 3R.
