# Supplementary material for: The Drosophila Enhancer of split Gene Complex: Architecture and Coordinate Regulation by Notch, Cohesin, and Polycomb Group Proteins
Source: G3 (Bethesda). 2013 Oct 1;3(10):1785–94. doi: 10.1534/g3.113.007534 (PMC3789803; doi:10.1534/g3.113.007534)
Supplement: Supporting Information [file supp_g3.113.007534_FigureS2.pdf]

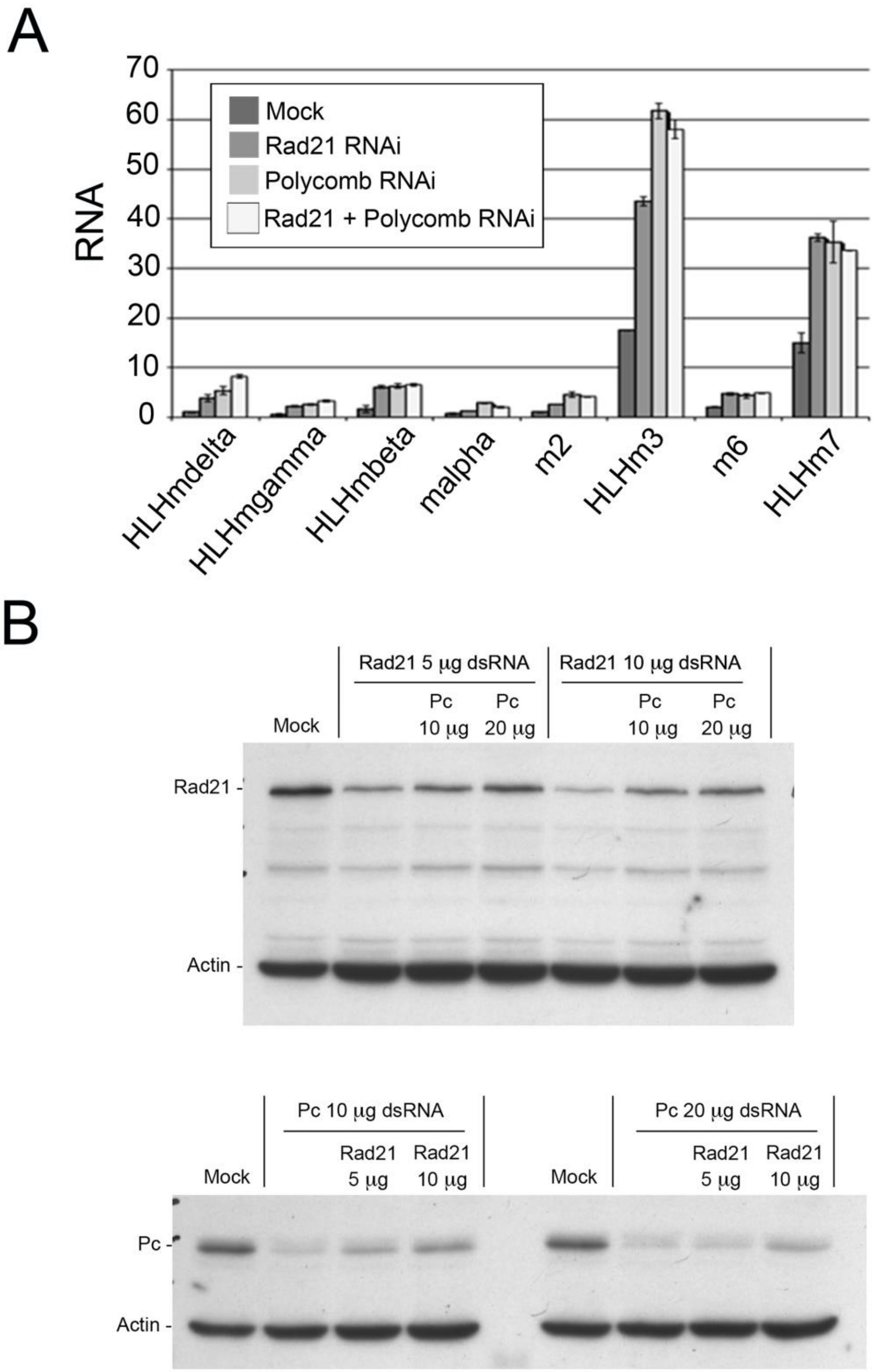

Figure S2 Simultaneous cohesin and PRC1 depletion does not synergistically increase E(spl)-C expression in BG3 cells. (A) E(spl)-C RNA levels in control BG3 cells, and BG3 cells depleted for Rad21, Pc, or both were measured by RT-qPCR as described in Figure 1. The BG3 cells were treated for five days with 5 μg of Rad21 dsRNA, 10 μg Pc dsRNA, or 5 μg of Rad21 plus 10 μg of Pc dsRNA per well. (B) The western blot shows extent of Rad21 and Pc protein depletion after five days of treatment with the indicated combinations of Rad21 and Pc dsRNA per well.
