# Supplementary material for: The Drosophila Enhancer of split Gene Complex: Architecture and Coordinate Regulation by Notch, Cohesin, and Polycomb Group Proteins
Source: G3 (Bethesda). 2013 Oct 1;3(10):1785–94. doi: 10.1534/g3.113.007534 (PMC3789803; doi:10.1534/g3.113.007534)
Supplement: Supporting Information [file supp_g3.113.007534_FigureS3.pdf]

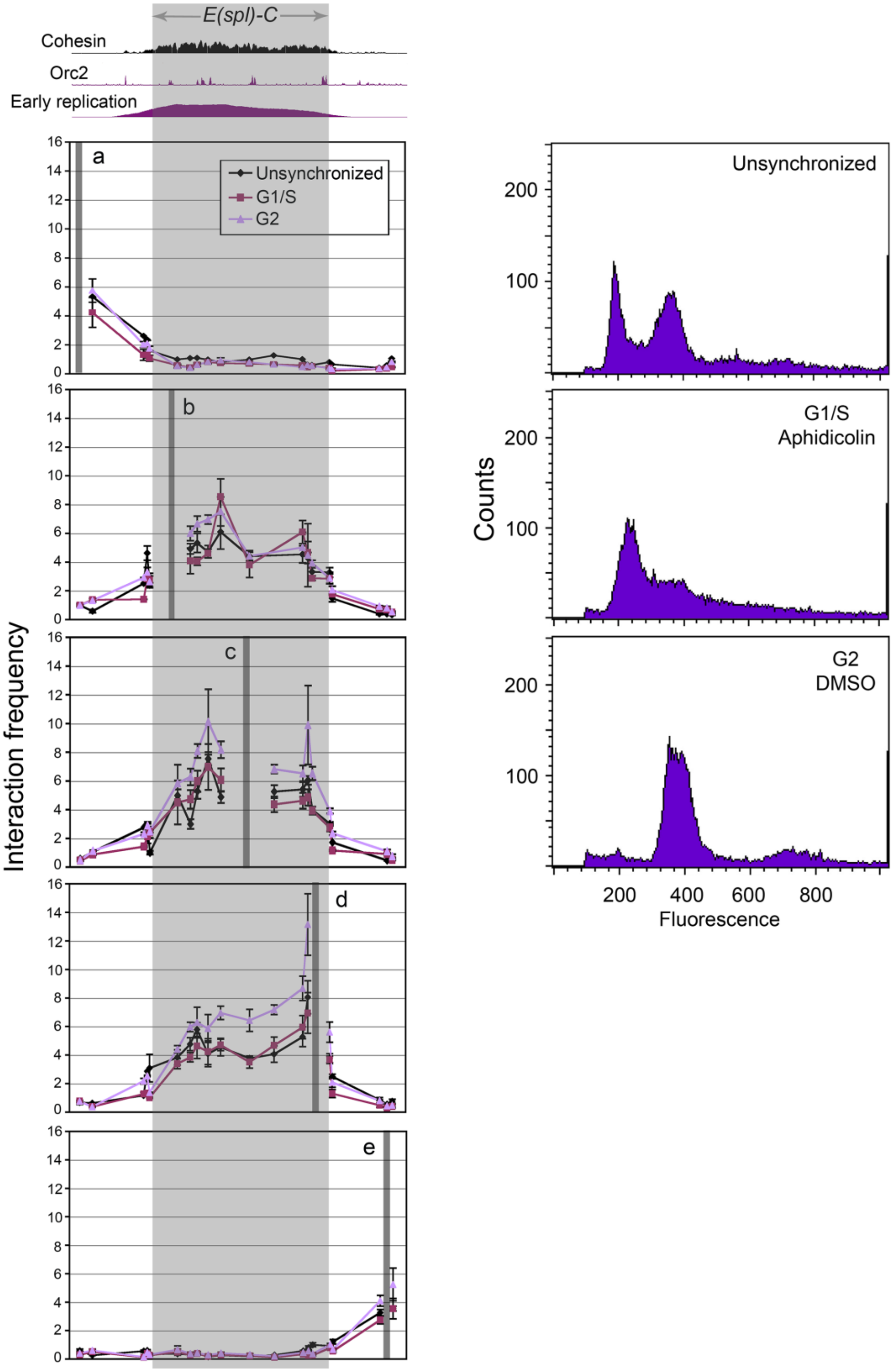

Figure S3 The higher order structure of the E(spl)-C is independent of the cell cycle stage in BG3 cells. The left panels compare the 3C analysis of control BG3 cells from Figure 1 to the 3C analysis of BG3 cells blocked at the G1/S boundary by treatment with 5 µg per mL aphidicolin for 26 hours, and BG3 cells blocked in G2 by treatment with 3% DMSO for 26 hours. The FACS analysis showing the cell cycle stages for each sample are shown in the panels on the right.
