# Supplementary material for: The Drosophila Enhancer of split Gene Complex: Architecture and Coordinate Regulation by Notch, Cohesin, and Polycomb Group Proteins
Source: G3 (Bethesda). 2013 Oct 1;3(10):1785–94. doi: 10.1534/g3.113.007534 (PMC3789803; doi:10.1534/g3.113.007534)
Supplement: Supporting Information [file supp_g3.113.007534_FigureS4.pdf]

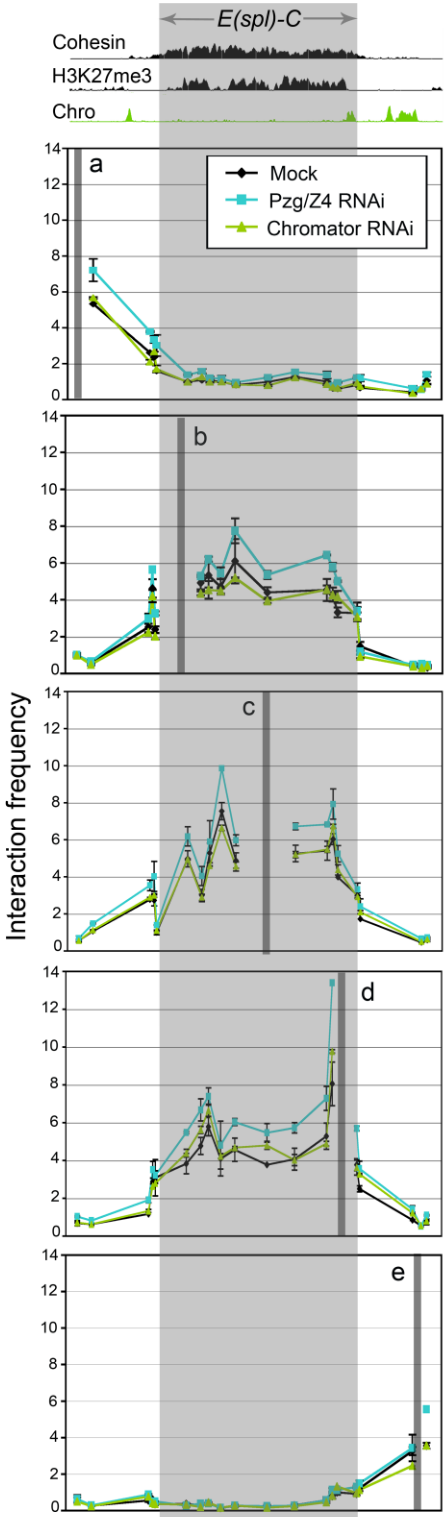

Figure S4 The higher order structure of the E(spl)-C is independent of the Chromator-Pzg/Z4 protein complex in BG3 cells. The panels compare the 3C analysis of control BG3 cells from Figure 1 to 3C analysis of BG3 cells treated with 40 µg of Pzg/Z4 or Chro dsRNA per well for six days. The Pzg/Z4 protein depletion is shown in Figure S5.
