# Supplementary material for: The Drosophila Enhancer of split Gene Complex: Architecture and Coordinate Regulation by Notch, Cohesin, and Polycomb Group Proteins
Source: G3 (Bethesda). 2013 Oct 1;3(10):1785–94. doi: 10.1534/g3.113.007534 (PMC3789803; doi:10.1534/g3.113.007534)
Supplement: Supporting Information [file supp_g3.113.007534_FigureS5.pdf]

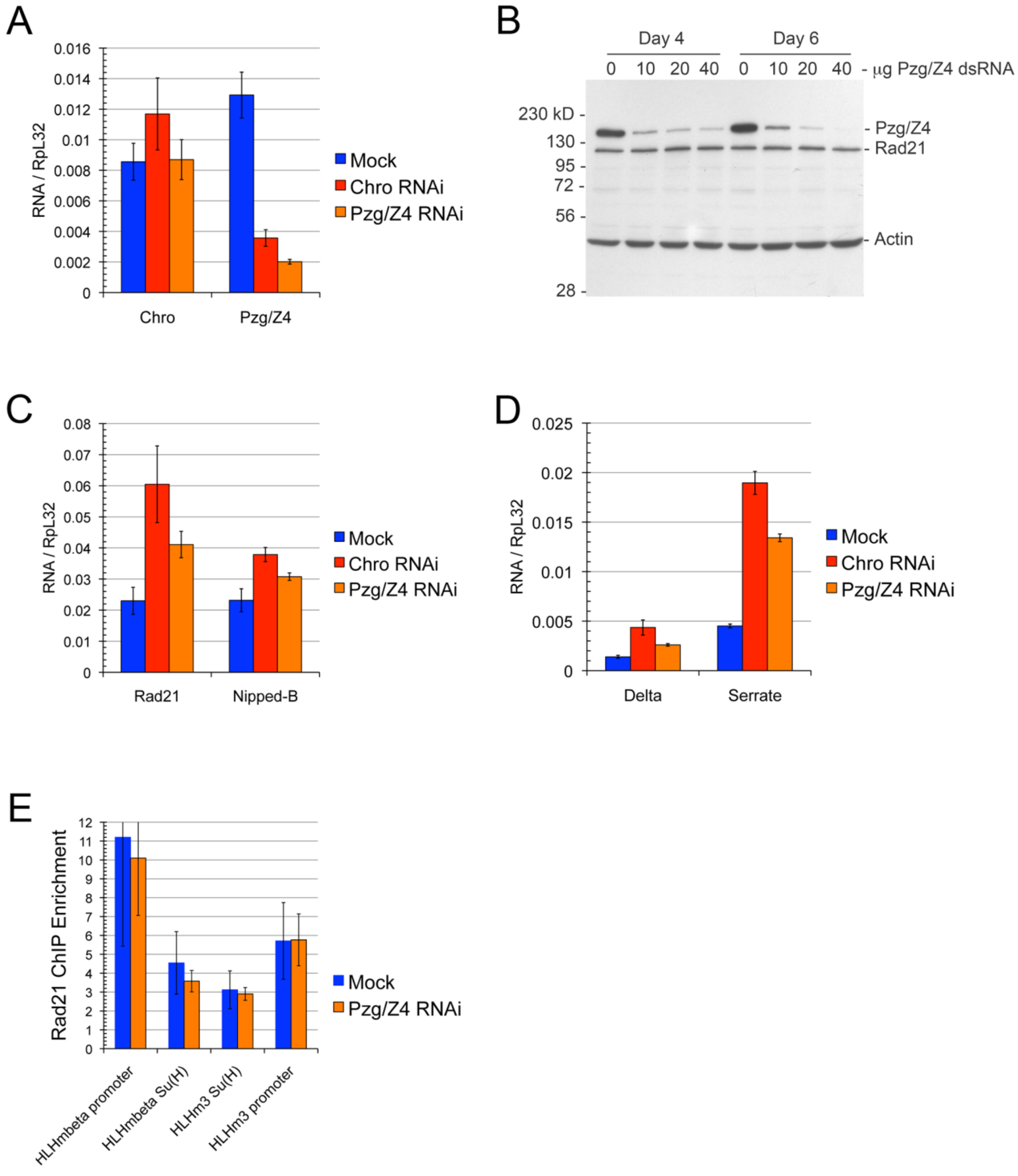

Figure S5 Depletion of the Chromator-Pzg/Z4 complex increases cohesin and Notch ligand gene expression in BG3 cells. (A) Chro and Pzg/Z4 RNAi treatment (40 µg dsRNA per well) for six days decreases Pzg/Z4 RNA levels. (B) The western blot shows the reduction of Pzg/Z4 protein levels with the indicated Pzg/Z4 dsRNA levels for the indicated time of treatment. (C) Chro and Pzg/Z4 RNAi treatment for six days increases *Rad21* and *Nipped-B* RNA levels. Panel B shows that Rad21 protein levels do not show a corresponding increase. (D) Chro and Pzg/Z4 RNAi treatment increase *Delta* and *Serrate* RNA transcripts. (E) Pzg/Z4 depletion does not alter cohesin (Rad21) binding to *HLHmb* and *HLHm3* genes in the E(spl)-C.
